# Nicotinamide phosphoribosyltransferase is a molecular target of potent anticancer agents identified from phenotype-based drug screening

**DOI:** 10.1038/s41598-019-43994-x

**Published:** 2019-05-23

**Authors:** Daisuke Yamaguchi, Takamichi Imaizumi, Kaori Yagi, Yuichi Matsumoto, Takayuki Nakashima, Akiyo Hirose, Naomi Kashima, Yukino Nosaka, Tomoko Hamada, Katsuya Okawa, Yoichi Nishiya, Kazuo Kubo

**Affiliations:** 1 Small Molecule Drug Research Laboratories, Research Functions Unit, R&D Division, Kyowa Hakko Kirin Co., Ltd., 1188, Shimotogari, Nagaizumi-cho, Sunto-gun, Shizuoka, 411-8731 Japan; 2 Research Core Function Laboratories, Research Functions Unit, R&D Division, Kyowa Hakko Kirin Co., Ltd., 1188, Shimotogari, Nagaizumi-cho, Sunto-gun, Shizuoka, 411-8731 Japan; 3 Clinical Sciences Research Laboratories, Translational Research Unit, R&D Division, Kyowa Hakko Kirin Co., Ltd., 1188, Shimotogari, Nagaizumi-cho, Sunto-gun, Shizuoka, 411-8731 Japan; 4 Fuji Research Park, R&D Division, Kyowa Hakko Kirin Co., Ltd., 1188, Shimotogari, Nagaizumi-cho, Sunto-gun, Shizuoka, 411-8731 Japan; 5 Corporate Social Responsibility Management Department, Kyowa Hakko Kirin Co., Ltd., 1-9-2, Ote-machi, Chiyoda-ku, Tokyo, 100-0004 Japan; 6 R&D Planning Department, R&D Division, Kyowa Hakko Kirin Co., Ltd., 1-9-2, Ote-machi, Chiyoda-ku, Tokyo, 100-0004 Japan; 7 Corporate Strategy & Planning Department, Kyowa Hakko Kirin Co., Ltd., 1-9-2, Ote-machi, Chiyoda-ku, Tokyo, 100-0004 Japan; 8 Open Innovation Department, R&D Division, Kyowa Hakko Kirin Co., Ltd., 3-6-6, Asahi-machi, Machida-shi, Tokyo, 194-8533 Japan; 9 Department of Biosciences & Informatics, Faculty of Science and Technology, Keio University, 3-14-1, Hiyoshi, Kohoku-ku, Yokohama-shi, Kanagawa, 223-8522 Japan

**Keywords:** Mechanism of action, Target identification

## Abstract

Phenotypic screening in drug discovery has been revived with the expectation of providing promising lead compounds and drug targets and improving the success rate of drug approval. However, target identification remains a major bottleneck in phenotype-based drug discovery. We identified the lead compounds K542 and K405 with a selective inhibition of cell viability against sphingosine-1-phosphate lyase 1 (SGPL1)-transduced ES-2 cells by phenotypic screening. We therefore performed an *in vivo* pharmacological examination and observed the antitumor activity of K542 in an HT-1080 tumor-bearing mouse xenograft model. SGPL1 was expected to be a therapeutic target in some cancers, suggesting that these lead molecules might be promising candidates; however, their mechanisms of action still remain unexplained. We therefore synthesized the affinity probe Ind-tag derived from K542 and identified the proteins binding to Ind-tag via a pull-down experiment. Proteomics and biochemical analyses revealed that the target molecule of these lead compounds was Nicotinamide phosphoribosyltransferase (NAMPT). We established K542-resistant DLD-1 and HT-1080 cells, and genetic analyses of these cells identified a missense mutation in the NAMPT-encoding gene. This enzymatic experiment clearly showed that K393 exerts enzymatic inhibition against NAMPT. These proteomics, genetics and biochemical analyses clarified that compounds K542 and K405 were NAMPT inhibitors.

## Introduction

Many pharmaceutical companies have struggled with phenotypic drug discovery (PDD) to deliver first-in-class small molecule drugs and succeeded in launching them in various therapeutic areas. Although target identification and the deconvolution of drugs is a considerable challenge, PDD is an effective strategy for screening campaigns targeting incompletely understood diseases or the disease-relevant cellular context.

Sphingolipids are bioactive mediators regulating various physiological functions, such as inflammation, immune response, cell proliferation and cell death^1,2^. Ceramide, a sphingolipid, is a key molecule in the sphingolipid metabolism and is also well known as a “tumor-suppressing lipid”^3,4^. Sphingosine-1-phosphate (S1P) is a ceramide metabolite generated in the ceramide-catabolic pathway that functions as a pro-tumor lipid promoting various cancer-supportive mechanisms, such as cellular transformation, proliferation, inhibition of apoptosis, angiogenesis and inflammation^2,5,6^.

Sphingosine-1-phosphate lyase 1 (SGPL1) catalyzes the irreversible degradation of S1P and functions as a proapoptotic enzyme in the ceramide-catabolic pathway^6^. SGPL1 was shown to promote apoptosis via the activation of p53 and p38 and was downregulated in intestinal adenomas of *Apc*^*Min*/+^ mice^7^. In contrast, the expression of SGPL1 was upregulated in ovarian carcinoma tissue^8^. We also confirmed the high expression of SGPL1 in some ovarian cancer cell lines through an in-house bioinformatics analysis of public gene expression databases^9^ (Supplemental Fig. S1a). The knock-down of SGPL1 induced greater cytotoxicity against the highly SGPL1-expressing ovarian cancer cell line, RMG-I, in comparison to the poorly SGPL1-expressing cell line ES-2 (Supplemental Fig. S1b).

Thus, we performed cell-based screening using an in-house chemical library to discover SGPL1 targeting compound in the context of cancer and identified the benzofuran analog K405 and the indole analog K542, which exerted cytotoxic activity against SGPL1-transduced ES-2 cells and highly SGPL1-expressing RMG-I cells. Unfortunately, the inhibition of SGPL1 by these compounds was not demonstrated by a SGPL1 enzyme assay. Phenotypic screening often identifies drugs with a rational molecular mechanism of action (MMoA); however, in some cases, phenotypic screening also identifies compounds with entirely unanticipated MMoAs, like our lead compounds and YM155^10^. To elucidate these MMoAs, we attempted to identify K542-targeting molecules by multiple approaches, especially chemoproteomic and chemogenetic approaches.

## Results

### Discovery of benzofurane/indole analogs selectively killing highly SGPL1-expressing cancer cells

**Figure 1:**
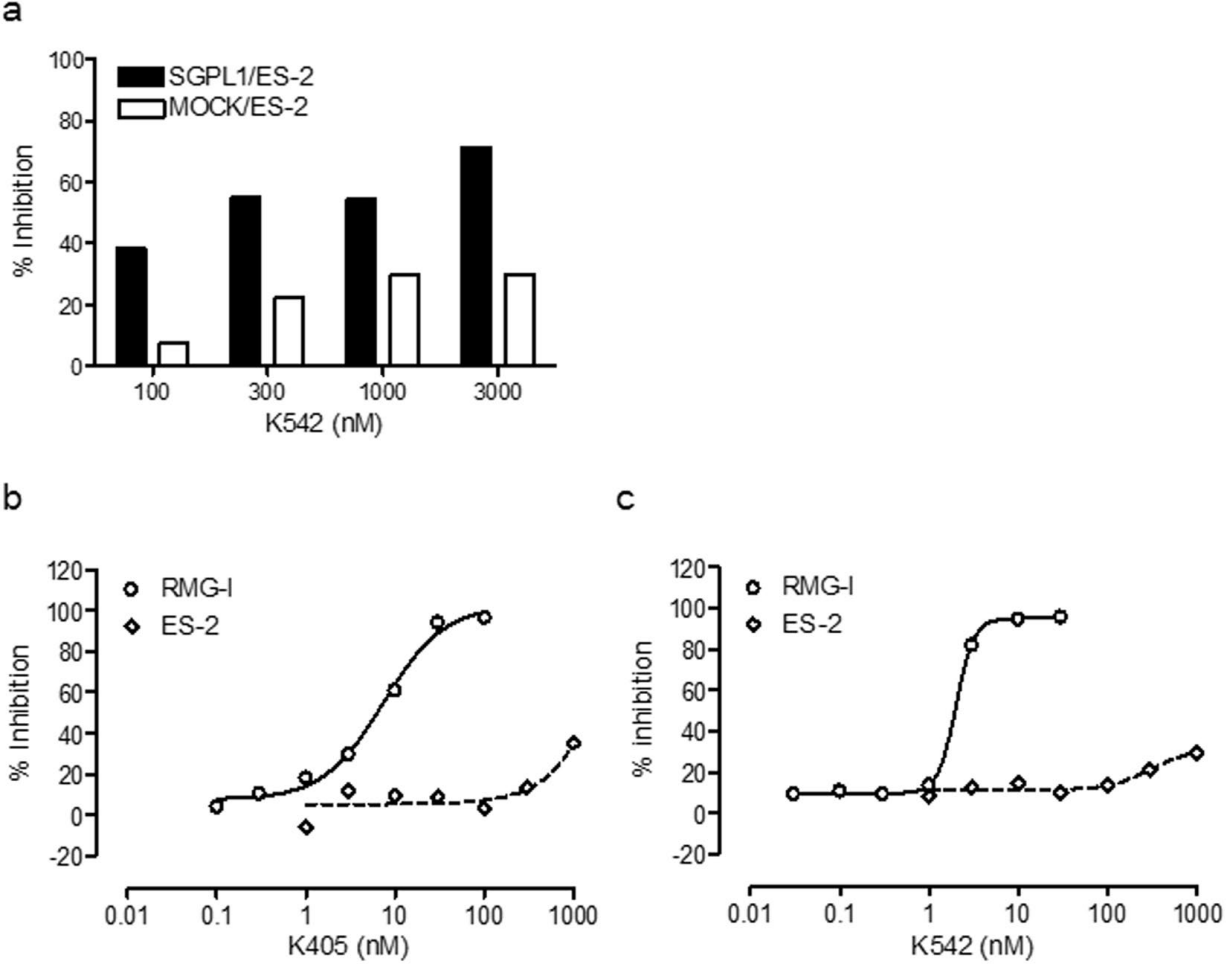
Novel synthetic compounds exerted potent *in vitro* anticancer activity against highly SGPL1-expressing cells. (**a**) Semi-dose-response *in vitro* anticancer activity of K542 against exogenous SGPL1-expressing ES-2 (Filled Bar) and empty vector-transfected MOCK/ES-2 (Open Bar). (**b**,**c**) Dose-response curves of the *in vitro* anticancer activity of K405 (**b**) and K542 (**c**) against endogenous highly SGPL1-expressing RMG-I (Open circle) and poorly SGPL1-expressing ES-2 (open square). Data indicate the mean, *n* = 6.

To identify SGPL1 inhibitors, we performed phenotypic screening of an in-house chemical library based on the results of a cell prliferation assay and indetified K405 and K542, which are benzofurane (Bnz) and indole (Ind) analogs, respectively. K542 exerted selective inhibition of cell viability against SGPL1-transduced ES-2 cell but not against the MOCK-transduced cell in the presence of ceramide (Fig. 1a). Ceramide potentiated the cytotoxic activity of SGPL1 siRNA on SGPL1-transduced ES-2 cells (Data not shown). Random screening was therefore performed in the presence of ceramide to sensiteize SGPL1-transduced ES-2 cells against pharmacological SGPL1 inhibition. To confrim the *in vitro* anticancer activity of the two lead compounds against cancer cells with a high or poor endogenous expression of SGPL1, we used RMG-I and ES-2 cells (Supplemental Fig. S1a). Both lead compounds exerted potent activity (GI_50_ value) against highly SGPL1-expressing RMG-I ovarian carcinoma cell but not poorly SGPL1expressing ES-2 cells (Fig. 1b,c, Tables 1 and 2). Activation of Caspase-3/7 was induced with single digit nanomolar K542 at 72 h after drug treatment, suggesting that the *in vitro* anticancer activity was triggered by caspase-3/7-mediated apoptosis (Data not shown). To investigate the structure-activity relationship (SAR), we evaluated the *in vitro* anticancer activity of various Bnz and Ind analogs. K405 and K542 were structurally similar and shared a common 3-pyridylethyl group at the position of the 2-amide side chain of the benzofuran ring and indole ring, respectively. Its replacement with an imidazoylpropyl (K393 and K216), 4-pyridylethyl (K142 and K543) or 3-pyridylmethyl (K143 and K541) group was tolerated. However, further extension with a propyltriazol moiety (K141 and K270) eliminated the *in vitro* anticancer activity. These results indicate that the basic moiety (*i*.*e*., the pyridyl or imidazoyl group) was favorable for potent cytotoxicity against RMG-I, and that these lead compounds targeted the same protein.

**Table 1:** Structure-activity relationship and affinity probe synthesis of benzofuran analog.

Compound	R^2^	R^7^	R^2′^	R^6′^	RMG-I GI_50_ (nM)	ES-2 GI_50_ (nM)

K405					20	>1000
K142					24	>1000
K143					16.9	>1000
K391					>1000	>1000
K393					0.8	>1000
K141					>1000	>1000
K710					690	>1000
K150					7.4	>1000
K461					120	>1000
Bnz-tag					N.T.^a^	N.T.^a^

^a^Not Tested.

**Table 2:** Structure-activity relationship and affinity probe synthesis of indole analog.

Compound	R^1^	R^2^	R^5′^	RMG-I GI_50_ (nM)	ES-2 GI_50_ (nM)

K542				1.8	>1000
K543				82	>1000
K541				0.7	>1000
K216				<1	>1000
K270				>300	>1000
K336				>1000	>1000
K209				6.1	>1000
Ind-tag				N.T.^a^	N.T.^a^

^a^Not Tested.

### Chemical proteomics identified NAMPT as a target of orphan anticancer Ind/Bnz analogs

We also tested the *in vitro* SGPL1 enzymatic assay to clarify the SGPL1 enzymatic inhibition of lead compounds. The lead molecules did not display enzymatic inhibition (Supplemental Fig. S2). Thus, we attempted the target identification of these lead compounds, as K542 is a exhibits highly potent *in vitro* anticancer activity.

To elucidate its molecular target by a pull-down experiment, we substituted the propyltriazol moiety at some positions of Bnz and Ind analogs using Huisgen cycloaddition to design affinity probes. The replacement of the 7-position of the benzofuran ring with a 3-(4-propyl-1-tirazolyl)-propyl ether group (K710) markedly reduced the cytotoxity against RMG-I cells. The modification of the 6′-position of the imidazopyridine ring of the Bnz analog with the 4-(4-propyl-1-triazolyl)methylphenyl moiety (K461) slightly decreased the activity. In contrast, the replacement of the 2′-position with a 4-propyl-1-triazolylmethyl group (K150) was well tolerated. Thus, we transformed the Bnz-tag at the 2′-position with alkynyl-PEG-FLAG by Huisgen cycloaddition^11,12^. We performed a pull-down experiment using the affinity probe Bnz-tag. No significant binding proteins were isolated despite using an excessive amount of Bnz-tag (Supplemental Fig. S3a). These data suggested that the modification of the 2′-position with a bulky epitope tag abrogated the target binding.

**Figure 2:**
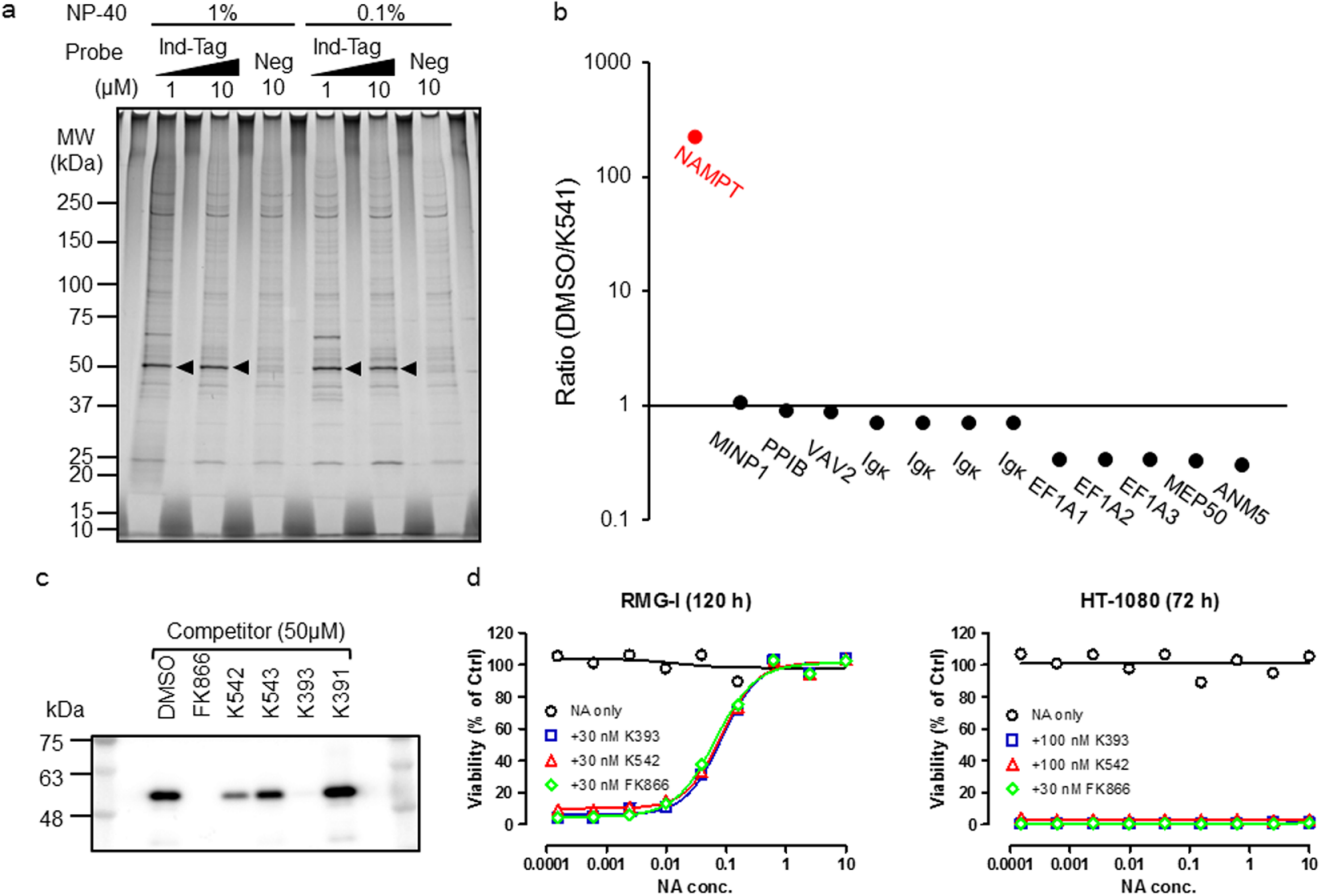
Identification of NAMPT by chemical pull-down experiment using an Ind-Tag. (**a**) Ind-Tag binding proteins were separated on SDS-PAGE and subjected to silver staining. RMG-I cell lysate was incubated with positive (Ind-tag) and negative (Neg) probes at the indicated concentrations. The band indicated with an arrowhead was cut from the gel and digested with trypsin. The pull-down experiments, which were conducted under similar conditions were independently performed with consistent results. (**b**) The ion intensity ratio of the identified Ind-tag binding proteins. The Ind-tag-specific binding protein is indicated with a red symbol. Lysate was pre-incubated with K541 at 10 μM and the pull-down experiments were performed using 1 μM Ind-tag. The Ind-tag binding proteins were identified by comparative LC-MS/MS. (**c**) A competitive pull-down assay using Ind-Tag. RMG-I cell lysate was pre-incubated with various compounds, and then NAMPT was precipitated with Ind-tag. NAMPT was separated on SDS-PAGE and detected by immunoblotting with anti-NAMPT antibody. (**d**) The cell viability curves of NAPRTase-proficient RMG-I cells and NAPRTase-deficient HT-1080 cells in the co-presence of a range of doses of NA and NAMPT inhibitors at the indicated concentrations. Cell viability was measured using CellTiter-Glo. Data indicate the mean, *n* = 3.

Accordingly, we newly synthesized the affinity probe Ind-tag by substituting the 5′-position with triazolyl-PEG-FLAG, as the 4-propyl-1-triazolylmethyl substitution of 5′-position (K209) was tolerated while the modification of the 1-position on the imidazole ring with the 3-(*tert*-butyldimethylsilyloxy)propyl moiety (K336) was not. The binding proteins were isolated from the RMG-I lysate using an Ind-tag and separated on SDS-PAGE. A significant binder was observed around a molecular weight of 50 kDa (Fig. 2a, Supplemental Fig. S3b). This protein stably bound to the Ind-tag under a high concentration of detergent NP-40.

We analyzed the 50 kDa protein band by mass fingerprinting (Table 3) by matrix-assisted laser desorption/ionization time-of-flight mass spectrometry (MALDI-TOF/MS) coupled with in-gel tryptic digestion (Supplemental Fig. S4). Furthermore, we also performed quantitative proteomics on Orbitrap velos by comparing the results of the K542-pretreated experiment with those of the DMSO-pretreated experiment. We succeeded in identifying Nicotinamide phosphoribosyltransferase (NAMPT) as a molecular target of K542. Interestingly, the significant binding protein, which was markedly competed out by an excess amount of the competitor K541, was merely NAMPT. Despite the presence of a competitor, all other proteins nonspecifically bound to the affinity probe Ind-tag (Fig. 2b).

**Table 3:** Peptide sequence identified as NAMPT.

Position	*m/z*		Missed cleavage	Sequence
Start –End	Observed	Expected		
190–196	907.4641	907.4568	0	LHDFGYR
118–127	1084.6298	1083.6225	0	AVPEGFVIPR
470–478	1144.5773	1143.5700	1	SYSFDEIRK
108–117	1184.6476	1183.6403	0	YDGHLPIEIK
479–491	1459.7378	1458.7305	0	NAQLNIELEAAHH
175–189	1714.8583	1713.8510	0	YLLETSGNLDGLEYK
85–99	1912.8664	1911.8591	1	DVYKEHFQDDVFNEK
197–216	1972.9964	1971.9891	0	GVSSQETAGIGASAHLVNFK
171–189	2140.0554	2139.0481	1	ILAKYLLETSGNLDGLEYK

Next, we carried out a competitive pull-down experiment to investigate the correlation between the *in vitro* anticancer activity and the competitive activity against NAMPT. The most potent compound K393 competed out the Ind-tag binding of NAMPT at a compound concentration of 50 µM, just like the well-known NAMPT inhibitor FK866, and K542 exhibited remarkable competition. In contrast, an inactive Bnz analog K391 did not show any competition and the moderately active Ind analog K543 showed moderate competition (Fig. 2c, Supplemental Fig. S5). Taken together, these results showed a good correlation between the cytotoxity and the probe-binding competition and also strongly suggested that active Ind/Bnz analogs target NAMPT. Nicotinic acid phosphoribosyltransferase (NAPRTase) converts nicotinic acid (NA) to nicotinic acid mononucleotide, a precursor of NAD^+^, and can ameliorate the NAD^+^-depletion by NAMPT inhibitor. Thus, NAD^+^ production depends on the NAMPT-mediated salvage pathway in NAPRTase-deficient cells. The cellular potency of K542 and K393 for HT-1080 were significantly reduced with NA co-treatment the same as known NAMPT inhibitors FK866 and GNE-617 for the NAPRTase-deficient HT-1080 cell line^13^, but not for the NAPRTase-proficient RMG-I cell line (Fig. 2d). To clarify the NAMPT enzymatic inhibition, we performed a fluorometric enzyme assay in the presence of a Bnz analog, K393 (Supplemental Fig. S6). An enzymatic inhibition assay indicated that the Ind/Bnz analogs are novel NAMPT inhibitors with a different chemotype from the known inhibitors FK866, GMX-1778, GNE-617 and CB30865^13–16^. These results demonstrated that Ind/Bnz analogs specifically target NAMPT and induce cell death by depleting NAD^+^.

### Acquired-resistance cells possess a missense mutation in the NAMPT gene

**Figure 3:**
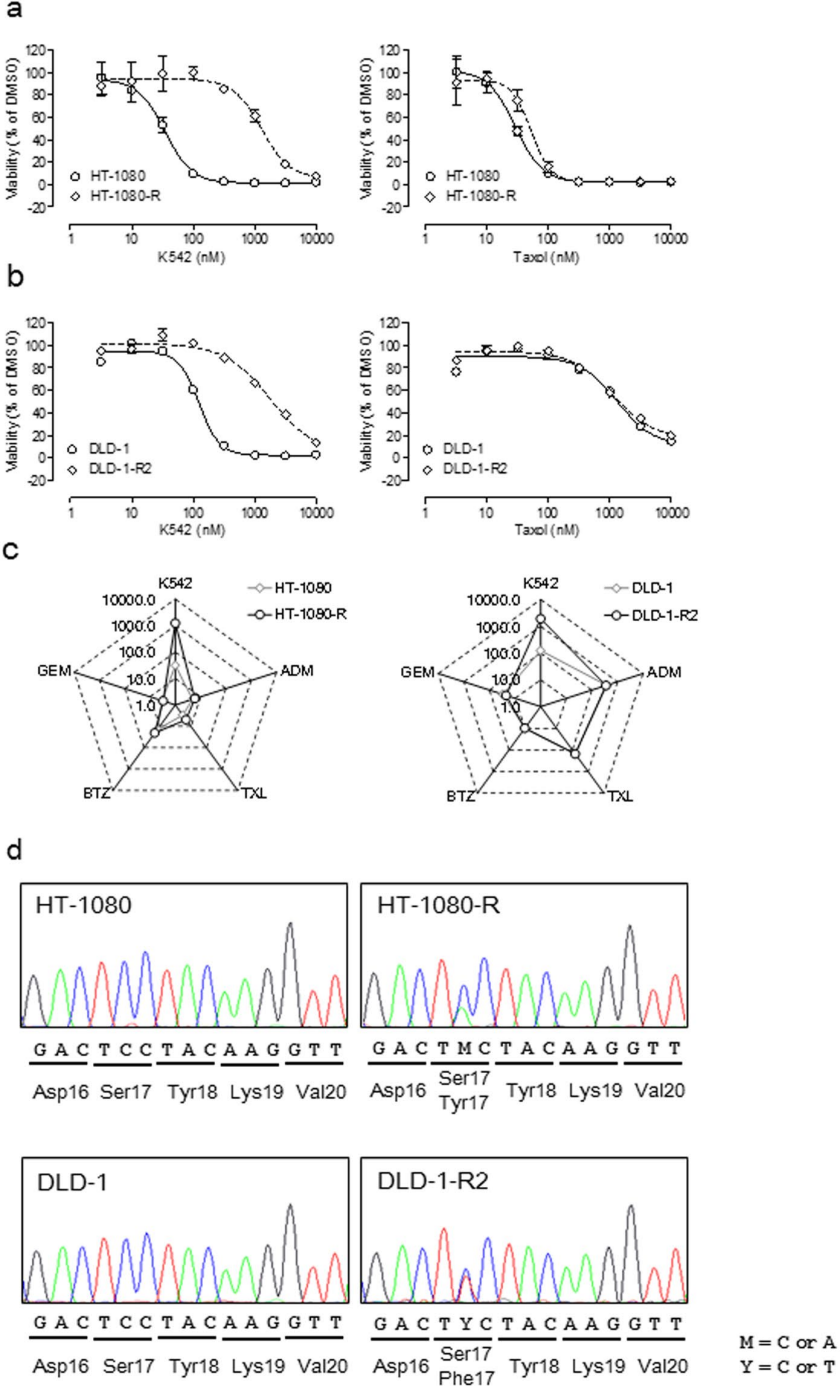
NAMPT gene mutation in K542 cell lines with acquired resistance. (**a**,**b**) Cell viability curves of K542-resistant HT-1080 (HT-1080-R) and its parent cell line (**a**) and K542-resistant DLD-1 (DLD-1-R2) and its parent cell line (**b**) in the presence of K542 or Taxol. Data indicate the mean ± s.e.m., *n* = 3. (**c**) A radar chart showing the IC_50_ value (nM) of K542 and some anticancer drugs against K542-resistant cells and their parent cells. (**d**) The nucleotide sequence of *NAMPT* cDNA of K542-resistant cells and their parent cells. The nucleotide symbol “M” is A or C and “Y” is C or T.

For the chemogenetic analysis, we established HT-1080 and DLD-1 cells with acquired resistance (HT-1080-R and DLD-1-R2, respectively) that were capable of growing in the presence of K542. We chose these cell lines based on the following rationale: HT-1080 is a NAPRTase-deficient cell line that requires NAMPT for NAD^+^ production, and DLD-1 is a MutS homolog 6 (MSH6)-lacking carcinoma cell line that can easily develop drug resistance. Both HT-1080-R and DLD-1-R2 showed sensitivity against chemotherapeutic agents, the same as their respective parent cells. As expected, these resistant cell lines showed specific and remarkable resistance to the NAMPT inhibitor K542 (Fig. 3a,b**)**. The IC_50_ value of resistant cells was one order of magnitude higher than that of the parent cells **(**Fig. 3c, Table 4).

**Table 4:** IC_50_ values and IC_50_ ratio of K542-resistant HT-1080 and DLD-1 cell lines.

Compound	HT-1080			DLD-1		
	WT^e^ IC_50_ (nM)	R IC_50_ (nM)	Ratio^f^	WT^e^ IC_50_ (nM)	R2 IC_50_ (nM)	Ratio^f^
K542	31	1200	39	120	1900	16
ADM^a^	4.8	5.9	1.2	380	360	0.95
TXL^b^	3.0	4.9	1.6	150	150	1.0
GEM^c^	3.3	3.1	0.93	30	22	0.74
BTZ^d^	14	21	1.5	10	19	1.0

^a^Adriamycin.

^b^Taxol.

^c^Gemcitabine.

^d^Bortezomib.

^e^Parent cells of each cell lines.

^f^Ratio of Resistant IC_50_/Parent IC_50._

To analyze the nucleotide sequence of the NAMPT gene by Sanger sequencing, we performed reverse-transcription into the cDNA library using mRNA extracted from the resistant cancer cell lines and their parent cell lines. We confirmed the heterozygous missense mutation in both HT-1080-R and DLD-1-R2 at nucleotide position 50, which encoded Ser17 in the parent cells. These missense mutations substituted the Tyr residue in HT-1080-R and the Phe residue in DLD-1-R2 for the Ser residue (Fig. 3d). NAMPT is a homodimeric enzyme, and its catalytic pocket forms between the interfaces of two molecules. Tyr18, which neighbors Ser17, is an important residue for the binding of NAMPT inhibitors containing the 3-amino-pyridine moiety by forming a pi-pi stacking interaction between its pyridine ring and the side chains of Tyr18 and Phe193 on another molecule^17–20^. K542 also possess the 3-pyridyl group and is expected to bind to NAMPT with the same binding mechanism.

To confirm whether or not the Tyr17/Phe17-substitution of Ser17 in NAMPT abrogated the binding to the Ind-Tag, we performed a pull-down experiment using the acquired-resistance cell lines. The binding of NAMPT expressed in mutant cells was significantly reduced (Supplemental Fig. S7). Thus, the substitution of bulky residues, such as Tyr or Phe, for the Ser17 residue can interrupt this pi-pi stacking formation, resulting in resistance to K542.

### K542 exerts *in vivo* antitumor activity in a xenograft model

**Figure 4:**
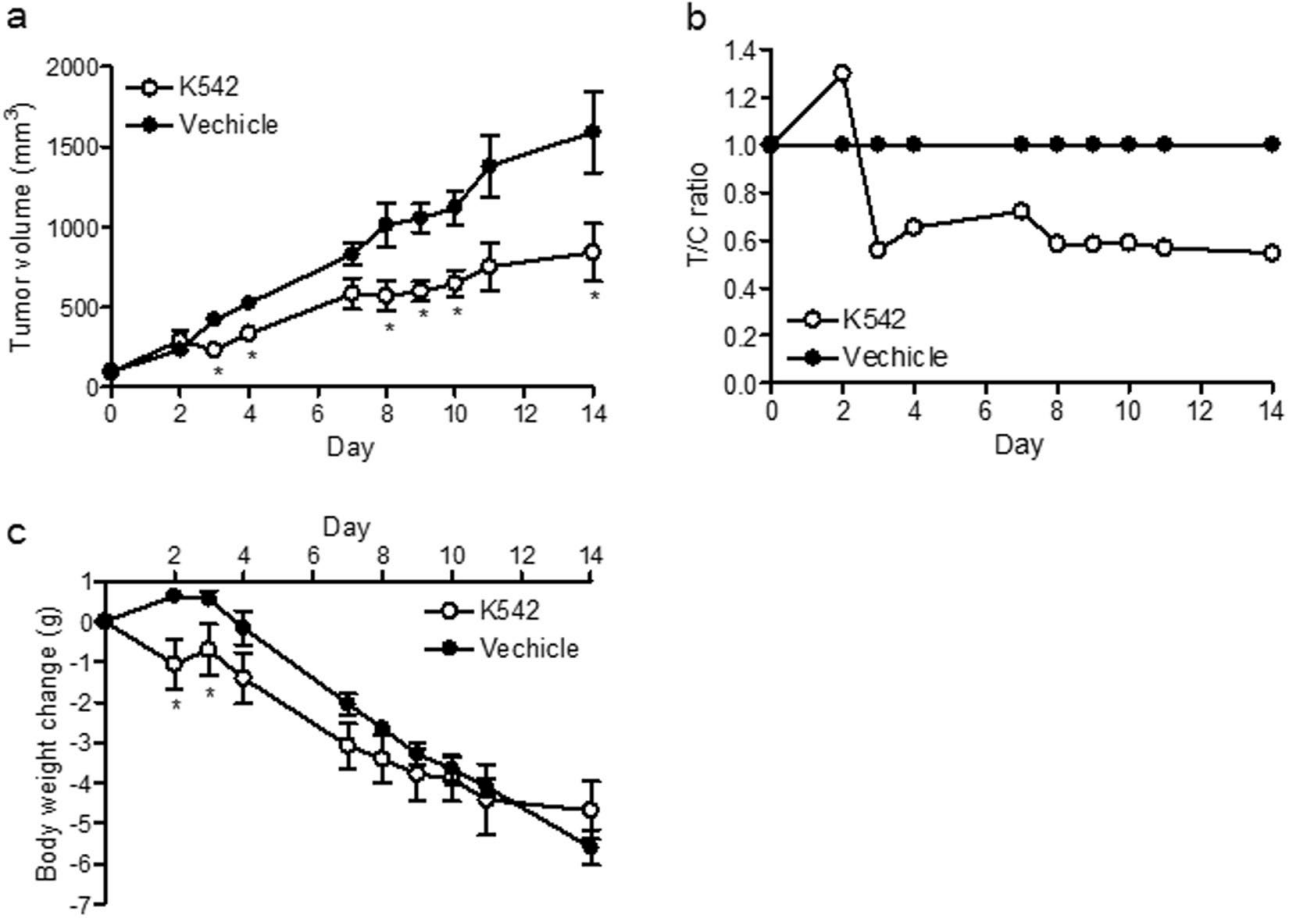
Antitumor activity of K542 in Xenograft model. (**a**) Tumor volume, (**b**) T/C ratio and (**c**) body weight change in HT-1080 xenograft models orally treated with vehicle or K542 (30 mg/kg, twice daily) for 12 days. Data indicate the mean ± s.e.m., *n* = 5. *p < 0.05, Wilcoxon’s rank sum test.

The SAR study (Tables 1 and 2) showed that the SAR of the Ind and Bnz analogs correlated well with each other. We therefore considered that highly potent K542 or K393 were both suitable for animal experiments from a potency perspective. However, we decided to use only K542 for *in vivo* antitumor testing because K393 did not dissolve well in administration solvent MC400. We observed the significant inhibition of tumor growth when K542 was orally administered twice daily to immunodeficient nude mice bearing NAPRTase-deficient HT-1080 tumors. We assessed the antitumor activity (Fig. 4a) based on the treated/control tumor volume ratio (T/C). The minimum T/C after 14 days of initial administration was 0.545 (Fig. 4b). To ensure the tolerability of K542 treatment, we evaluated the body weight change with the same regimen. The body weight slightly decreased in the early phase of treatment (days 2–3). However, at the end of the regimen, there was no significant difference in the weight loss in comparison to after vehicle treatment (Fig. 4c).

## Discussion

In this study, we identified the pharmacological target of Bnz and Ind analogs, which exhibit potent antiproliferative activity *in vitro* (Fig. 1b,c). Only one protein with a molecular weight of approximately 50 kDa specifically bound to the affinity probe Ind-tag derived from Ind analog K542 and was isolated by a pull-down experiment (Fig. 2a). Focused proteomics coupled with in-gel tryptic digestion and non-labeled comparative shot-gun proteomics clarified that the protein was NAMPT, a rate-limiting enzyme in the salvage NAD^+^ biosynthesis pathway^21^. We confirmed the relationship between the cytotoxity activity and the NAMPT-binding of Bnz/Ind analogs by a competitive pull-down assay following immunoblotting. Since K393 exhibited the enzymatic inhibition of NAMPT, and NA co-treatment reduced the antiproliferative activity against NAPRTase-proficient RMG-I cells by K542 and K393, similarly to the known NAMPT inhibitor, FK866, we hypothesized that these lead compounds specifically target NAMPT (Fig. 2d, Supplemental Fig. S6).

We established cells with acquired-resistance to K542 and clearly showed drug-specific resistance to K542 but not to other chemotherapeutic drugs (Fig. 3c, Table 4). A genetic analysis of K542-resistant cell lines revealed a missense mutation in Ser17, a residue adjacent to the key residue Tyr18, forming a pi-pi interaction with NAMPT inhibitor^17–19^. This finding suggests that the substitution of a bulky residue for Ser17 disarranged the pi-pi interaction between Tyr18 and the pyridine ring of the NAMPT inhibitor K542. A pull-down experiment using resistant cells demonstrated the hindrance of binding between the probe and NAMPT, supporting our hypothesis (Supplemental Fig. S7).

Pharmacological NAD^+^ depletion by an NAMPT inhibitor reportedly led to tumor cell death followed by ATP depletion^14,15^. K542 exerted a 30% reduction in the intratumoral NAD^+^ level (Supplemental Fig. S8a). It seemed to lead to tumor growth inhibition (T/C_min_ = 0.545) in an HT-1080 xenograft mouse model (Fig. 4a,b) by the same mode of action as other NAMPT inhibitors. O’Brien *et al*. reported that GNE-617 exerted a 95% depletion in the intratumoral NAD^+^ level and more potent antitumor activity than K542 in a NAPRTase-deficient HT-1080 xenograft model^13^. This discrepancy in effects between GNE-617 and K542 appeared to be due to differences in poor drug-exposure and/or the pharmacokinetic profile of K542 (Supplemental Fig. S8b) and/or the complexity of *in vivo* metabolic homeostasis—specifically, the NAD^+^ or NAD^+^ precursor was exogenously supplied to tumors from normal tissue or tumor adjacent stromal tissue.

NAD^+^ is an important metabolite that maintains tumor cells under various conditions of cellular response, such as genetic repair, apoptosis regulation, stress response, energy production and biomass production. IDH1-mutant glioma required NAMPT for the production of NAD^+^ and showed vulnerability to the inhibition of NAMPT, because mutant IDH1 reduces the expression of NAPRTase^22^. Hence, NAMPT-targeting therapy is a promising approach for IDH1 mutation harboring tumors.

In an *in vivo* study, significant weight loss was not observed, and K542 was well-tolerated (Fig. 4c), like GNE-617^13^; however, no other toxicological assessments were performed. Some clinical trials of the NAMPT inhibitors FK866, GMX1778 and its prodrug GMX1777 failed to demonstrate promising therapeutic efficacy, and further trials were discontinued due to the common dose-limiting toxicities of thrombocytopenia and gastrointestinal toxicity^17,23^. FK866, GMX-1778 and GNE-617 demonstrated human megakaryocyte toxicity in an *in vitro* colony forming unit-megakaryocyte assay, but NA supplementation mitigated the megakaryocyte toxicity induced by these inhibitors^24^. These results suggest that the co-administration of NA with NAMPT inhibitor may ameliorate thrombocytopenia. On the other hand, it was also reported that the co-administration of NA with GNE-617 did not increase the therapeutic window of GNE-617^13^. Collectively, these findings suggest that NAMPT-targeting tumor therapy does not benefit patients as expected, and the clarification of the complicated tumor metabolism is necessary for the clinical development of drugs targeting the tumor metabolism.

Finally, through several chemical biological approaches, including a pull-down assay, a competitive assay and an enzymatic inhibition assay, we showed that synthetic chemicals with highly potent anticancer activity targeted the rate-limiting enzyme NAMPT in the NAD^+^ biosynthesis salvage pathway. Furthermore, genetic analyses of cells with acquired resistance clarified the missense mutation in the NAMPT gene, and this mutation was assumed to result in the impairment of the pi-pi interaction between the Tyr18 residue and the 3-aminopyridyl ring of NAMPT inhibitor.

## Methods

### Antibodies and chemicals

Mouse Anti-NAMPT monoclonal antibody (OMNI379), PBEF/NAMPT rabbit monoclonal antibody (D7V5J) and SGPL1 antibody (AF5535) were purchased from AdipoGen (San Diego, CA, USA), Cell Signaling Technology (Danvers, MA, USA) and R&D systems (Minneapolis, MN, USA), respectively. FK-866 (sc-205325A), Adriamycin (046-21521), Taxol (T7402), Gemcitabine (Gemzal® Injection 200 mg) and C6 Ceramide (860506P) were purchased from Santa Cruz Biotechnology, Wako, Sigma-Aldrich, Eli-Lilly and Avanti Polar Lipids, respectively. Bortezomib was synthesized in house. The detailed methods for synthesizing the Ind/Bnz analogs are provided in the Supplemental Information.

### Cell culture

Human ovarian clear cell carcinoma ES-2 cells were purchased from American Type Culture Collection (ATCC, Manassas, VA, USA) and maintained in MaCoy’s 5A medium (Life Technologies, Carlsbad, CA, USA) supplemented with 10% (v/v) heat-inactivated fetal bovine serum (FBS; SAFC Biosciences, Lenxa, KS, USA) and 1% (v/v) penicillin-streptomycin (PS) (Life Technologies). Human ovarian mesonephroid adenocarcinoma RMG-I cells were purchased from Japanese Collection of Research Bioresources (JCRB, Osaka, Japan) and maintained in Ham’s F12 medium (Life Technologies) supplemented with 10% (v/v) heat-inactivated FBS (Life Technologies) and 1% (v/v) PS. Human sarcoma HT-1080 cells were purchased from JCRB and maintained in minimal essential medium (Life Technologies) supplemented with 10% (v/v) heat-inactivated FBS, 1% (v/v) non-essential amino acids (Life Technologies), 2% (v/v) sodium bicarbonate (final concentration, 0.15%; Life Technologies) and 1% (v/v) PS. Human colon adenocarcinoma DLD-1 cells were purchased from JCRB and maintained in RPMI-1640 medium (Life Technologies) supplemented with 10% (v/v) heat-inactivated FBS, 1% (v/v) HEPES (Life Technologies) (final 20 mM), 1% (v/v) sodium pyruvate (Life Technologies), 0.62% (v/v) glucose (Sigma-Aldrich, St. Louis, MO, USA) and 1% (v/v) PS. Cells were incubated at 37 °C in an air-conditioned atmosphere with 5% CO_2_.

### *In vitro* anticancer activity assay

A WST-8 assay was performed using CCK-8 according to the manufacturer’s protocol. ES-2 cells and SGPL1/ES-2 cells were seeded with MaCoy’s 5 A medium containing 10% (v/v) FBS, 1% (v/v) PS and 0.3 μM ceramide in a 384-well flat-bottom plate at 500 and 200 cells/well, respectively. After 16 h of culturing, cells were treated with drugs diluted with MaCoy’s 5A medium containing FBS and PS at the indicated concentrations. The cell number was quantified using CCK-8 by measuring the absorbance with an EnVision 2103 Multilabel Reader microplate reader (PerkinElmer, Waltham, MA, USA) after 5 days of culturing. The inhibition of cell viability was calculated using the equation presented below. RMG-I cells were seeded with Ham’s F12 medium containing 10% (v/v) FBS, 1% (v/v) PS and 0.3 μM ceramide in a 384-well flat-bottom plate at 2500 cells/well. After 16 h of culturing, cells were treated with drugs diluted with Ham’s F12 medium containing FBS and PS at the indicated concentrations. The cell number was quantified using CCK-8, and then inhibition of cell viability was calculated using the following equation:

$${\rm{Inhibiton}}\,( \% )=\{1-\frac{({\rm{Absorbance}}\,{\rm{of}}\,{\rm{drug}}\,{\rm{treatment}}-{\rm{Absorbance}}\,{\rm{of}}\,{\rm{blank}})}{({\rm{Absorbance}}\,{\rm{of}}\,{\rm{DMSO}}\,{\rm{treatment}}-{\rm{Absorbance}}\,{\rm{of}}\,{\rm{blank}})}\}\times 100$$

For the cell viability assay in the presence of NA, HT-1080 and RMG-I cells were seeded into a 96-well flat-bottom plate at 1000 and 2000 cells/well, respectively. Approximately 24 h after seeding, cells were treated with NA, which had been serially diluted from 10 μM at a ratio of 1/4 and indicated concentrations of NAMPT inhibitors. At 72 h or 120 h after treatment, the number of cells was quantified using CellTiter-Glo (Promega, Madison, WI, USA) by measuring the absorbance with a SPECTRAmax340PC-384 microplate reader (Molecular Devices, Sunnyvale, CA, USA). Control cells were treated with the same concentrations of vehicle (DMSO alone), and no other substances. To calculate the cell viability, the cell viability of DMSO-treated cells and medium alone was calculated as 100% and 0%, respectively.

For the cell viability assay of K542-resistant cells, HT-1080, DLD-1 and our K542-resistant cells were seeded into a 96-well flat-bottom plate at 500 or 2200 cells/well. Approximately 24 h after seeding, the cells were treated with drugs that had been serially diluted at a ratio of 1/√10. At 72 h after treatment, the number of cells was quantified using CCK-8 by measuring the absorbance with a SPECTRAmax340PC-384 microplate reader. Control cells were treated with DMSO at the same concentrations. To calculate the cell viability, the cell viability of DMSO-treated cells and medium alone was calculated as 100% and 0%, respectively. The IC_50_ value of viability was calculated with a four-parameter logistic model using the XLfit ver. 4.2 (IDBS, Guildford, Surrey, UK) and GraphPad Prism 4 (GraphPad, La Jolla, CA, USA) software programs.

### Pull-down isolation of proteins binding to the affinity probe

Sub-confluent RMG-I cells adhering to a 10- or 15-cm culture dish were washed with ice-cold DPBS, lysed in 1 to 2 mL of lysis buffer on ice for 30 min, and then harvested using a cell scraper. The lysis buffer was 20 mM HEPES-NaOH (pH 7.3 ± 0.2) containing 150 mM NaCl, the appropriate concentration of NP-40, and Complete Mini EDTA-free protease inhibitor cocktail (Roche, Mannheim, Germany). The cell lysate was centrifuged at 18,000 × *g* for 20 min to exclude cell debris and insoluble matter. The cleared lysate was incubated with 1 or 10 μM affinity probe at ambient temperature for 2 h. These mixtures were mixed with 10 μL of Anti-FLAG^®^ M2-Magnetic beads (Sigma-Aldrich) and rotated at ambient temperature for 15 or 30 min or at 4 °C overnight. Supernatants were discarded by collecting beads on a magnetic stand. The collected beads were then washed with lysis buffer three times and rinsed with 20 mM HEPES-NaOH containing 150 mM NaCl (HBS) once. Binding proteins were eluted by mixing in a solution (50 μl) of 150 μg/mL 3xFLAG^®^ peptide dissolved with HBS. Eluted aliquots were denatured with 20% (v/v) Non-Reducing Lane Marker Sample buffer (Thermo Scientific, Rockford, IL, USA) and 5% (v/v) Bond-Breaker™ TCEP solution (Thermo Scientific) by boiling at 95 °C for 5 min and separated on SDS-PAGE at 20 mA of constant current for 90 min based on Laemmli’s method. Gels were stained with a PlusOne™ silver staining kit (GE Healthcare, Little Chalfont Buckinghamshire, England). Images of silver-stained gels were captured using an ImageQuant LAS 4000 and the ImageQuant TL software program (GE Healthcare).

### In-gel tryptic digestion and mass spectrometry

Mass spectrometric identification of proteins was performed as previously described^25^. In brief, proteins were excised separately from gels, followed by in-gel digestion with trypsin (V5111; Promega) in a buffer containing 50 mM ammonium bicarbonate (pH 8.0) (Sigma-Aldrich) overnight at 37 °C. Molecular mass analyses of the tryptic peptides were performed by MALDI-TOF/MS using an ultrafleXtreme (Bruker Daltonics, Billerica, MA, USA). Proteins were identified by comparing the molecular weights determined by MALDI-TOF/MS to the theoretical peptide masses of the proteins registered in NCBInr.

### Proteomic analyses

Trypsin digestion of the pulled-down proteins was performed by referring to the manual of the In-Solution Tryptic Digestion and Guanidination Kit (Thermo Scientific). The digestion was stopped with trifluoroacetic acid (TFA) and then desalted with C-tips (AMR Inc., Tokyo, Japan). After centrifugal concentration, the peptides were dissolved in 10 μl of 2% acetonitrile/0.1% TFA. The peptides were analyzed with an LTQ Orbitrap Velos (Thermo Scientific) equipped with Advance CaptiveSpray SOURCE (Michrom BioResources, Auburn, CA, USA). The samples were then separated with Acclaim PepMap 100 C18 75 μm × 15 cm nanoViper using an UltiMate 3000 Nano LC System (Thermo Scientific). Buffer A (0.1% formic acid in water) and buffer B (0.1% formic acid in acetonitrile) were used as the mobile phases for gradient separation. The gradient was 95 min from 5% B to 45% B and was maintained for 10 min for washing. The flow rate was set at 300 nL/min. Data were acquired over the 300–2,000 m/z range with the data-dependent scan mode (resolution, 60,000).

The top five peptides were subjected to MS/MS for fragmentation. Each sample was analyzed in triplicate (2 μl/injection). Raw files were analyzed by Proteome Discoverer 1.3 for protein identification from human proteome sequences (Uniprot) supplemented with 3 × FLAG and mouse IgG_1_. The search parameters were set as follows: Enzyme Trypsin, Minimum peptide length 6, Maximum missed cleavage 2, Fixed modifications Carbamidomethyl (C), Variable modifications Oxidation (M), Peptide tolerance 10 ppm, MS/MS tolerance 0.8 Da, false discovery rate at peptide level 0.05. Label-free quantitation of each peptide was performed with SIEVE 2.1 with the default parameters, except for MS/MS, which was required to construct frames. Peptides with more than one peptide sequence match were used for quantitation. The protein ratio was obtained by averaging the ratios of all quantified peptides. Possible contaminants from the environment were excluded from the results.

### The competitive pull-down assay

RMG-I cell lysate was treated with various competitors at the shown concentrations, and then NAMPT was isolated by a pull-down assay, as described above. Isolated NAMPT was separated on SDS-PAGE and transferred to a PVDF membrane by Western blotting, as described below. Semi-dry Western blotting was performed with Tris/CAPS buffer containing 15% methanol (anode buffer) and Tris/CAPS buffer containing 0.02% SDS (cathode buffer) at 70 mA of constant current for 50 min. NAMPT was detected with anti-NAMPT antibody (Cell Signaling Technologies) and HRP-conjugated secondary antibody using SuperSignal West Dura Chemiluminescence substrate (Thermo Scientific).

### The establishment of acquired-resistance cells

DLD-1 and HT-1080 cells were continuously exposed to K542 for several months. At an early point, most cells died. The remaining living cells were then continuously cultured in the presence of K542. The concentration of K542 was gradually escalated to a concentration 10-fold greater than the IC_50_ value for cell viability. Finally, cells proliferating in the presence of high concentrations of K542 were established.

### The genetic analyses of NAMPT inhibitor-resistant cell lines

The NAMPT cDNA fragment was amplified from the cDNA library of resistant cells by a PCR using KOD-plus-neo DNA polymerase (TOYOBO, Osaka, Japan) and the primers NAMPT_F2 and qNAMPT_R1. The amplified cDNA fragment was concentrated by ethanol precipitation, separated by electrophoresis, and harvested from an agarose gel. The nucleotide sequence of NAMPT was analyzed with an ABI3100 genetic analyzer (Applied Biosystems, Foster City, CA, USA) using a BigDye terminator v3.1 cycle sequencing kit.

### The HT-1080 tumor-bearing mouse xenograft model

All animal studies were performed in accordance with the Standards for Proper Conduct of Animal Experiments at Kyowa Hakko Kirin Co., Ltd., under the approval of the company’s Institutional Animal Care and Use Committee. Fuji Research Park of Kyowa Hakko Kirin Co., Ltd., is fully accredited by the Association for the Assessment and Accreditation of Laboratory Animal Care, International.

Five-week-old male CAnN.Cg-Foxn1 <nu> /CrlCrlj(BALB-nu/nu) mice (nude mice) were purchased from Charles River Laboratories. All mice were provided sterilized food and water during the term of the examination. For antitumor activity tests, nude mice were subcutaneously inoculated into the hind flank region with 3 × 10^6^ cells of HT-1080 in a 100-μL cell suspension of DPBS. On the seventh day after inoculation, tumor-bearing mice were randomized into two groups (n = 5 per group) based on their average tumor volume. The average volumes were 97.59 ± 4.50 mm^3^ for vehicle treatment and 95.30 ± 4.71 mm^3^ for K542 treatment on the day of treatment initiation (day 0). Mice were orally administered a vehicle solution (0.5% [w/v] methylcellulose 400 solution; Wako, Osaka, Japan) or K542 at 30 mg/kg (0.5% [w/v] methylcellulose 400 solution) twice a day for 12 days. The tumor volume was measured using digital calipers on days 0, 2, 3, 4, 7, 8, 9, 10, 11 and 14. The body weight was also measured on the same day as the tumor volume was determined. The antitumor effect was evaluated by calculating the T/C value with the following equation: T/C = (V/V_o_ for the K542-treated group)/(V/V_o_ for the vehicle-treated group). Where V and V_0_ are the tumor volume on each day of evaluation and day 0, respectively. The tumor volume was calculated with the equation 1/2 × (length of major axis) × (length of minor axis)^2^ and was statistically evaluated by Wilcoxon’s rank sum test using the SAS software program (version 9.2; SAS Institute Inc., Cary, NC, USA).

## Supplementary information

Supplemental information

## Data Availability

The datasets generated and analyzed during the current study are available from the corresponding author on reasonable request.
