# Supplementary material for: Nicotinamide phosphoribosyltransferase is a molecular target of potent anticancer agents identified from phenotype-based drug screening
Source: Sci Rep. 2019 May 23;9:7742. doi: 10.1038/s41598-019-43994-x (PMC6533267; doi:10.1038/s41598-019-43994-x)
Supplement: Supplementary file 1 — Supplemental information [file 41598_2019_43994_MOESM1_ESM.doc]

**Supplemental information for:**

**Nicotinamide phosphoribosyltransferase is a molecular target of potent anti-cancer agents identified from phenotype-based drug screening**

**Authors:**

Daisuke Yamaguchi^1,*^, Takamichi Imaizumi^1^, Kaori Yagi^5^, Yuichi Matsumoto^7^, Takayuki Nakashima^2^, Akiyo Hirose^8^, Naomi Kashima^3^, Yukino Nosaka^2^, Tomoko Hamada^1^, Katsuya Okawa^4^, Yoichi Nishiya^1^, Kazuo Kubo^6, 9^

**Affiliations:**

^1^Small Molecule Drug Research Laboratories, Research Function Unit, R&D Division,

^2^Research Core Function Laboratories, Research Function Unit, R&D Division,

^3^Clinical Sciences Research Laboratories, Translational Research Unit, R&D Division,

^4^Fuji Research Park, R&D Division,

Kyowa Hakko Kirin Co., Ltd. 1188, Shimotogari, Nagaizumi-cho, Sunto-Gun, Shizuoka, 411-8731, Japan

^5^Corporate Social Responsibility Management Department,

^6^R&D Planning Department, R&D Division,

Kyowa Hakko Kirin Co., Ltd. 1-9-2, Otemachi, Chiyoda-ku, Tokyo, 100-0004, Japan

^7^Immunology & Allergy Research Laboratories, Immunology & Allergy R&D Unit, R&D Division,

^8^Open Innovation Department, R&D Division,

Kyowa Hakko Kirin Co., Ltd. 3-6-6, Asahi-machi, Machida-shi, Tokyo, 194-8533, Japan

^9^Department of Biosciences & Informatics, Faculty of Science and Technology,

Keio University, 3-14-1 Hiyoshi, Kohoku-ku, Yokohama-shi, Kanagawa 223-8522, Japan

^*^Correspondence: [daisuke.yamaguchi@kyowa-kirin.co.jp](mailto:daisuke.yamaguchi@kyowa-kirin.co.jp)

**Supplemental Methods**

*siRNA transfection*

SGPL1 siRNA (SI03085551, QIAGEN, Hilden, Germany), KIF11 siRNA (SI00064855, QIAGEN) and Negative control siRNA (SI03650318) were reverse-transfected with HiPerFect reagent (QIAGEN), after which 3 μL of 2 μM siRNA was mixed with 0.8 μL of HiPerFect and 21.2 μL OPTI-MEM (Life Technologies) in each well of a 96-well flat-bottom plate. RMG-I and ES-2 cell suspensions (175 μL) were seeded onto siRNA/HiPerFectin complex in a 96-well plate at 5000 and 1000 cells/well, respectively. After 168 h of incubation, the cell viability was determined using a Cell Counting Kit-8 (CCK-8; Dojindo, Kumamoto, Japan).

*The expression and purification of SGPL1*

Sf9 cells (Invitrogen, Carlsbad, CA, USA) were infected with a recombinant bacmid derived from a baculovirus encoding an N-terminal MBP-tag fused SGPL1 gene corresponding to amino acid Pro2-His568. The gene encoding the SGPL1 protein is based on Genbank entry NM_003901. The fragment was amplified by a polymerase chain reaction (PCR). The 5’- and 3’ fragments of human SGPL1 were amplified from human small intestine cDNA using 5’-fragment primer pair (SGPL-1 and SGPL-7) and 3’-fragment primer pair (SGPL-2 and SGPL-5), respectively. The cDNA fragment encoding Pro2-His568 was amplified from these 5’- and 3’-fragments using SGPL-1 and SGPL-2 as a primer pair, and then ligated in pFastBac1 (Invitrogen) engineered with MBP-tag. The expression cassette encoding MBP-fused SGPL1 was transposed into the bacmid in DH10Bac competent cells (Invitrogen). Bacmid was transfected into Sf9 cells (Invitrogen) with CELLfectin (Invitrogen) in Sf-900III (Invitrogen) medium according to the manufacturer’s protocol.

After three days of culturing, the culture medium was harvested as recombinant virus solution. Fresh Sf9 cell were infected with the harvested virus solution twice. The final culture medium of the infected cells was harvested, and the virus titer was determined by real-time PCR.

Sf9 cells were maintained in Sf-900III medium and infected with recombinant viruses at MOI of 3 in Sf-900III medium. After 2 days’ culture, cells were harvested by centrifugation and lysed with 75 ml of PBS containing 2% (w/v) Nonidet® P-40 (NP-40), 1 mM Dithiothereitol (DTT) and complete protease inhibitor. After rotation at 4°C for 60 min, the lysates were centrifuged. Cleared lysate was loaded onto an Amylose resin (New England Bio Labs, Ipswich, MA, USA) column (1.5 cm diameter × 3.5 cm length) equilibrated with 25 mM Tris-HCl (pH8.0) containing 200 mM NaCl, 2 mM 2-mercaptethanol (2-ME) and 2% (w/v) NP-40). After washing with the wash buffer 1 (25 mM Tris-HCl, pH8.0 containing 200 mM NaCl, 1% [w/v] NP-40 and 2 mM 2-ME) and wash buffer 2 (25 mM Tris-HCl, pH8.0 containing 200 mM NaCl and 2 mM 2-ME), the MBP-SGPL1 was eluted with 100 mL of linear gradient of maltose from 0 to 5 mM in wash buffer 2 Elution was performed at a flow rate of 0.5 mL/min, and fractions of 0.5 mL were collected. The MBP-SGPL1 in each fraction was separated and confirmed on SDS-PAGE. The fractions containing recombinant protein were concentrated with an YM-10 filter (Millipore, Burlington, MA, USA) and dialyzed with 25 mM Tris-HCl, pH8.0 containing 200 mM NaCl and 2 mM 2-ME for 16 h at 4°C. After dialysis, recombinant proteins were concentrated up to approximately 1 mL.

*SGPL1 enzyme assay*

The SGPL1 enzyme activity was monitored by a mobility shift assay. The assay buffer was 36 mM potassium phosphate buffer containing 36 mM NaF, 70 mM Sucrose, 3 mM DTT, 0.4 mM Pyridoxal 5’-phosphate and 0.6 mM EDTA. SGPL1 enzyme was diluted with assay buffer at 37.5 μg/mL. Sphingosine 1-phosphate fluorescein (S1P Fluorescein, S-200F; Echelon, Salt Lake City, UT, USA) was dissolved with MeOH at 0.1 mg/ml. The required amount of S1P Fluorescein/MeOH solution was dried up and dissolved with assay buffer containing 0.2% (v/v) Triton X-100 by brief sonication. DMSO solution of the compound was diluted with assay buffer. A 20-μL aliquot of the enzyme was incubated with 30 μL of S1P Fluorescein solution and 10 μL compound solution for 6 h. After incubation, the reaction was terminated with 15 μL of isopropanol containing 0.075% (w/v) Brij35 and 0.5% (v/v) Coating Reagent 3. The substrate and product were separated in separation buffer containing 0.5% (v/v) Coating Reagent 8 and 20% (v/v) isopropanol, and they were measured using a LabChip EZ reader (PerkinElmer).

*Establishment of SGPL1-stable transfectant*

SGPL1 cDNA was amplified by a PCR using Platinum Pfx DNA polymerase (Invitrogen), pcDNA/SGPL1 FW 2nd and pcDNA/SGPL1 RV 2nd primers and then ligated between the *Xba*I and *Kpn*I sites of pcDNA3.1/Hygro(-) (Invitrogen). This SGPL1-encoding vector and empty vector were transfected into ES-2 cells with Lipofectamine2000 for SGPL1/ES-2 and MOCK/ES-2, respectively. At 6 h post-transfection, FBS was added to the cultured cells, and after 24 h of culturing, the transfected cells were seeded into a 96-well plate at 100 cells/well and cultured in the presence of 0.3 mg/mL Hygromycin (Wako) to establish a stable transfectant. The high expression of SGPL1 in the clone was confirmed by Western blotting using an SGPL1 antibody (R&D Systems). These transfectants were maintained in the same manner as the parent ES-2 cells.

*Enzymatic assay*

The NAMPT enzymatic inhibition assay was performed using the CycLex NAMPT colorimetric Assay Kit (CY-1251; CycLex, Nagano, Japan) according to the manufacturer’s 2-Step Assay protocol. K393 and FK-866 was serially diluted with DMSO at a 50-fold final concentration. Nicotinamide was diluted with PBS at 1 mM and 2-Step Assay Buffer I was prepared according to the manufacturer’s protocol. In brief, 2 μL of recombinant NAMPT was incubated with 2 μL of 50-fold concentrated drugs, 10 μL of 1 mM nicotinamide and 6 μL of dH_2_O in 80 μL of 2-Steps Assay Buffer I for 60 min at 30°C after brief shaking. The 2-step Assay Buffer II was prepared according to the manufacturer’s protocol. A total of 20 μL of prepared Buffer II was added to wells and then incubated at 30°C for 20 min. The absorbance at 450 nm was measured with a microplate reader.

*Pharmacokinetics study*

BALB/cAnNCrlCrlj mice (Charles River Laboratories) were orally treated with a single dose of K542 at 30 mg/kg (n=2). At 0.5, 1, 2, 4, 7and 24 hour after administration, a 50-μL blood sample was collected from the tail vein using a heparin-coated capillary tube (Drummond Scientific) and centrifuged plasma was analyzed with API2000 liquid chromatography-tandem mass spectrometry (LC-MS/MS) system (AB Sciex, Framingham, MA, USA) to measure the K542 concentration in plasma.

*LC-MS/MS analyses of intratumoral NAD^+^*

For the LC-MS/MS analysis of intratumoral NAD^+^, nude mice were subcutaneously inoculated into the hind flank region with 3 × 10^6^ cells of HT-1080 in a 100-μL cell suspension of DPBS. Seven days after inoculation, tumor-bearing mice were randomized into two groups (n=3 per group) based on similar average tumor volumes. The average volumes were 186.85±7.34 mm^3^ for vehicle treatment and 186.32 ± 8.33 mm^3^ for K542 treatment at the initiation of treatment (day 0). Mice were orally administered a vehicle solution or K542 (30 mg/kg) twice a day for 4.5 days. At 8 h after the final administration, the tumor and spleen were resected from euthanized animals. The NAD^+^ level was statistically evaluated by Student’s *t*-test using the SAS software program.

The relative amount of NAD^+^ in the tumor and spleen was determined by LC-MS/MS analyses. Resected tissues were homogenized with Milli Q water at a four-fold volume of tissue weight using TissueLyser (QIAGEN). Then, 100 μL of 1 μM 2-Chloroadenosine was mixed as an internal control with 50 μL of homogenized sample. The mixture was centrifuged for 5 min at 5,000 × *g* at a temperature of 4°C. The cleared supernatant (75 μL) was mixed with an equal amount of MilliQ water. Obtained samples were separated using an ACQUITY-UPLC system (Waters, Milford, MA, USA) equipped with a PC HILIC column (5-μm, 4.6 mm I.D. × 50 mm; Shiseido, Kyoto, Japan) and analyzed using an API4000 coupled mass spectrometer (AB Sciex) equipped with a turbo spray interface in positive ionization mode. The LC-MS/MS data were acquired using the Analyst 1.4.2 software program (AB Sciex)

*Primers*

| **Primer** | **Sequence (5’-3’)** |
| --- | --- |
| Primer SGPL-1 | GGGGAGCGGAATTCCTGGAAGTTCTGTTCCAGGGGCCTAGCACAGACCTTCTGATGTTGAAGGCCTTTGA |
| Primer SGPL-2 | TAGAAAGGGTGGTACCTTAGTGGGGTTTTGGAGAACCATTCATCTGGCTGCCCTG |
| Primer SGPL-5 | CTATCAGTTCTTCGTCGATA |
| Primer SGPL-7 | TCTGACTTGAGGAAGCGAGC |
| pcDNA/SGPL1 FW 2^nd^ | CCTAGCACAGTCTAGACCACCATGCCTAGCACAGACCTTCTGATGTTGAAGGCC |
| pcDNA/SGPL1 RV 2^nd^ | ACCCCAAAACGGTACCTTAGTGGGGTTTTGGAGAACCATTCATCTG |
| NAMPT-F2 | TTTTTCTCCTTCCTCGCAGC |
| qNAMPT-R1 | TGTTGGGATCAGCAACTGGG |

*Chemical synthesis of compounds*

**Synthesis of K710*^a^***

*^a^*Reagents and conditions: (a) propionic acid, PPA, 80 °C; (b) hexamethylenetetramine, TFA, 80 °C; (c) ethyl 2-bromoacetate, KI, K_2_CO_3_, DMF, 100 °C; (d) NaBH_4_, EtOH, 0 °C; (e) SOCl_2_, CHCl_3_, 60 °C; (f) 2, NaH, DMF then 4 N NaOH aq., 0 °C -r.t.; (g) BBr_3_, CH_2_Cl_2_, 50 °C; (h) 3-(2-aminoethyl)pyridine, EDCI・HCl, HOBt・H_2_O, DMF, r.t.; (i) 1-bromo-3-chloropropane, K_2_CO_3_, acetone, 60 °C; (j) NaN_3_, DMA, 70 ^o^C; (k) 1-pentyne, CuSO_4_・5H_2_O, sodium L-ascorbate, 1,4-dioxane, H_2_O, r.t. K710: ^1^H-NMR (CDCl_3_) *δ*: 8.59–8.45 (m, 2H), 7.65–7.54 (m, 2H), 7.36–7.23 (m, 2H), 6.99 (s, 1H), 6.91 (s, 1H), 6.80 (s, 1H), 5.48 (s, 2H), 4.58 (t, *J* = 6.4 Hz, 2H), 4.10 (t, *J* = 5.6 Hz, 2H), 3.84–3.74 (m, 2H), 3.04 (t, *J* = 7.1 Hz, 2H), 2.80 (q, *J* = 7.6 Hz, 2H), 2.69–2.58 (m, 2H), 2.64 (s, 3H), 2.59 (s, 3H), 2.44–2.37 (m, 2H), 1.73–1.57 (m, 2H), 1.31 (t, *J* = 7.6 Hz, 3H), 0.92 (t, *J* = 7.4 Hz, 3H). LC-MS (ESI): *m*/*z* = 621 [M + H]^+^.

**Synthesis of Compound K150*^a^***

*^a^*Reagents and conditions: (a) glycolic acid, 150 °C; (b) TBSCl, imidazole, DMF, r.t.; (c) SOCl_2_, CHCl_3_, 60 °C; (d) 13, NaH, DMF then 4 N NaOH aq., 0 °C -r.t.; (e) 3-(2-aminoethyl)pyridine, EDCI・HCl, HOBt・H_2_O, DMF, r.t.; (f) MsCl, Et_3_N, CH_2_Cl_2_, 0 °C; (g) NaN_3_, DMA, 70 °C; (h) 1-pentyne, CuSO_4_・5H_2_O, sodium L-ascorbate, 1,4-dioxane, H_2_O, r.t. K150: ^1^H-NMR (CDCl_3_) *δ*: 8.50–8.48 (m, 2H), 7.58 (d, *J* = 7.8 Hz, 1H), 7.34 (s, 1H), 7.33 (s, 1H), 7.27–7.23 (m, 1H), 7.01 (s, 1H), 6.99 (s, 1H), 6.96 (s, 1H), 6.82 (t, *J* = 6.3 Hz, 1H), 5.68 (s, 2H), 5.54 (s, 2H), 3.95 (s, 3H), 3.74–3.62 (m, 2H), 2.96 (t, *J* = 7.3 Hz, 2H), 2.67 (s, 3H), 2.64 (s, 3H), 2.57 (t, *J* = 7.3 Hz, 2H), 1.63–1.52 (m, 2H), 0.90 (t, *J* = 7.3 Hz, 3H). LC-MS (ESI): *m*/*z* = 579 [M + H]^+^.

**Synthesis of K142, K393, K405, K391 and K143*^a^***

*^a^*Reagents and conditions: (a) amine, EDCI・HCl, HOBt・H_2_O, DMF, r.t. K142: ^1^H-NMR (CDCl_3_) *δ*: 8.54–8.52 (m, 2H), 7.36 (s, 1H), 7.17–7.15 (m, 2H), 6.95 (d, *J* = 1.5 Hz, 1H), 6.91 (s, 1H), 6.89 (d, *J* = 1.5 Hz, 1H), 6.82 (t, *J* = 6.2 Hz, 1H), 5.50 (s, 2H), 3.88 (s, 3H), 3.72 (td, *J* = 7.1, 6.2 Hz, 2H), 2.95 (t, *J* = 7.1 Hz, 2H), 2.83 (q, *J* = 7.5 Hz, 2H), 2.64 (s, 3H), 2.60 (s, 3H), 1.33 (t, *J* = 7.5 Hz, 3H). LC-MS (ESI): *m*/*z* = 484 [M + H]^+^. K393: ^1^H-NMR (CDCl_3_) *δ*: 7.52 (s, 1H), 7.37 (s, 1H), 7.08 (s, 1H), 6.98–6.96 (m, 2H), 6.92–6.90 (m, 2H), 6.80 (t, *J* = 6.2 Hz, 1H), 5.51 (s, 2H), 4.05 (t, *J* = 7.1 Hz, 2H), 3.90 (s, 3H), 3.48 (q, *J* = 6.6 Hz, 2H), 2.83 (q, *J* = 7.7 Hz, 2H), 2.64 (s, 3H), 2.60 (s, 3H), 2.17–2.07 (m, 2H), 1.33 (t, *J* = 7.7 Hz, 3H). LC-MS (ESI): *m*/*z* = 487 [M + H]^+^. K405: ^1^H-NMR (CDCl_3_) *δ*: 8.51–8.47 (m, 2H), 7.57 (d, *J* = 7.9 Hz, 1H), 7.36 (s, 1H), 7.27–7.21 (m, 1H), 6.95 (s, 1H), 6.91 (s, 1H), 6.88 (s, 1H), 6.81 (t, *J* = 5.1 Hz, 1H), 5.50 (s, 2H), 3.88 (s, 3H), 3.71 (td, *J* = 7.0, 5.1 Hz, 2H), 2.95 (t, *J* = 7.0 Hz, 2H), 2.83 (q, *J* = 7.6 Hz, 2H), 2.64 (s, 3H), 2.60 (s, 3H), 1.33 (t, *J* = 7.6 Hz, 3H). LC-MS (ESI): *m*/*z* = 484 [M + H]^+^. K391: ^1^H-NMR (CDCl_3_) *δ*: 8.56 (dd, *J* = 4.6, 1.6 Hz, 2H), 7.42 (s, 1H), 7.28–7.23 (m, 3H), 6.97 (d, *J* = 1.3 Hz, 1H), 6.92–6.89 (m, 2H), 5.50 (s, 2H), 4.65 (d, *J* = 6.3 Hz, 2H), 3.87 (s, 3H), 2.83 (q, *J* = 7.6 Hz, 2H), 2.64 (s, 3H), 2.60 (s, 3H), 1.33 (t, *J* = 7.6 Hz, 3H). LC-MS (ESI): *m*/*z* = 470 [M + H]^+^. K143: ^1^H-NMR (CDCl_3_) δ: 8.61 (s, 1H), 8.56-8.52 (m, 1H), 7.70 (d, *J* = 7.9 Hz, 1H), 7.40 (s, 1H), 7.30–7.12 (m, 2H), 6.95 (s, 1H), 6.92–6.87 (m, 2H), 5.50 (s, 2H), 4.65 (d, *J* = 6.3 Hz, 2H), 3.86 (s, 3H), 2.82 (q, *J* = 7.6 Hz, 2H), 2.64 (s, 3H), 2.60 (s, 3H), 1.32 (t, *J* = 7.6 Hz, 3H).LC-MS (ESI): *m*/*z* = 470 [M + H]^+^.

**Synthesis of K141*^a^***

*^a^*Reagents and conditions: (a) 3-chloropropylamine, EDCI・HCl, HOBt・H_2_O, DMF, r.t.; (b) NaN_3_, DMA, 100 °C; (c) 1-pentyne, CuSO_4_・5H_2_O, sodium L-ascorbate, 1,4-dioxane, H_2_O, r.t. K141: ^1^H-NMR (CDCl_3_) *δ:* 7.40 (s, 1H), 7.35 (s, 1H), 6.99 (t, *J* = 5.9 Hz, 1H), 6.95 (s, 1H), 6.93 (s, 1H), 6.90 (s, 1H), 5.52 (s, 2H), 4.42 (t, *J* = 6.6 Hz, 2H), 3.91 (s, 3H), 3.52–3.43 (m, 2H), 2.86 (q, *J* = 7.6 Hz, 2H), 2.67 (s, 3H), 2.67 (t, *J* = 7.6 Hz, 2H), 2.61 (s, 3H), 2.28–2.17 (m, 2H), 1.74–1.60 (m, 2H), 1.33 (t, *J* = 7.6 Hz, 3H), 0.94 (t, *J* = 7.2 Hz, 3H). LC-MS (ESI): *m*/*z* = 530 [M + H]^+^.

**Synthesis of K462*^a^***

*^a^*Reagents and conditions: (a) NBS, MeOH, r.t.; (b) SOCl_2_, CHCl_3_, 60 °C; (c) 21, NaH, DMF then 4 N NaOH aq. 0 ^o^C-r.t.; (d) 3-(2-aminoethyl)pyridine, EDCI・HCl, HOBt・H_2_O, DMF, r.t.; (e) 4-(hydroxymethyl)phenylboronic acid, Pd(dppf)Cl_2_, Cs_2_CO_3_, 1,4-dioxane, H_2_O, 100 °C; (f) MsCl, Et_3_N, DMA, 0 °C; (g) NaN_3_, DMA, 70 °C; (h) 1-pentyne, CuSO_4_・5H_2_O, sodium L-ascorbate, 1,4-dioxane, H_2_O, r.t. K462: ^1^H-NMR (CDCl_3_) *δ*: 8.52–8.46 (m, 2H), 7.58 (d, *J* = 7.8 Hz, 1H), 7.38 (s, 1H), 7.34 (d, *J* = 7.8 Hz, 2H), 7.30 (s, 1H), 7.28–7.24 (m, 1H), 7.22 (d, *J* = 8.8 Hz, 2H), 6.99 (s, 1H), 6.97 (s, 1H), 6.82 (t, *J* = 5.9 Hz, 1H), 5.58 (s, 2H), 5.53 (s, 2H), 3.93 (s, 3H), 3.76–3.68 (m, 2H), 2.96 (t, *J* = 6.8 Hz, 2H), 2.86 (q, *J* = 7.3 Hz, 2H), 2.72 (t, *J* = 7.3 Hz, 2H), 2.35 (s, 3H), 2.32 (s, 3H), 1.76–1.68 (m, 2H), 1.35 (t, *J* = 7.3 Hz, 3H), 0.98 (t, *J* = 7.8 Hz, 3H). LC-MS (ESI): *m*/*z* = 683 [M + H]^+^.

**Synthesis of K541 and K543*^a^***

*^a^*Reagents and conditions: (a) Boc_2_O, DMAP, CH_2_Cl_2_, r.t.; (b) NaBH_4_, EtOH, r.t.; (c) MsCl, LiCl, 2,4,6-Collidine, CH_2_Cl_2_, r.t.; (d) 2, NaH, DMF, r.t.; (e) LDA, THF, -78 °C then CO_2_ (solid), r.t.; (f) amine, EDCI・HCl, HOBt・H_2_O, DMF, r.t.; (g) 5N NaOH aq., EtOH, r.t. K541: ^1^H-NMR (CDCl_3_) *δ*: 9.27 (br s, 1H), 8.61 (d, *J* = 1.1 Hz, 1H), 8.55 (dd, *J* = 4.8, 1.1 Hz, 1H), 7.70 (d, *J* = 8.1 Hz, 1H), 7.35–7.22 (m, 3H), 7.11 (dd, *J* = 8.6, 1.6 Hz, 1H), 6.91 (s, 1H), 6.73 (d, *J* = 1.6 Hz, 1H), 6.65 (t, *J* = 5.9 Hz, 1H), 5.53 (s, 2H), 4.68 (d, *J* = 5.9 Hz, 2H), 2.80 (q, *J* = 7.5 Hz, 2H), 2.64 (s, 3H), 2.60 (s, 3H), 1.27 (t, *J* = 7.5 Hz, 3H). LC-MS (ESI): *m*/*z* = 439 [M + H]^+^. K543: ^1^H-NMR (CDCl_3_) *δ*: 9.17 (s, 1H), 8.54 (dd, *J* = 4.4, 1.8 Hz, 2H), 7.35–7.25 (m, 2H), 7.18–7.11 (m, 3H), 6.91 (s, 1H), 6.60 (s, 1H), 6.19 (t, *J* = 5.9 Hz, 1H), 5.54 (s, 2H), 3.79–3.71 (m, 2H), 2.94 (t, *J* = 7.0 Hz, 2H), 2.79 (q, *J* = 7.6 Hz, 2H), 2.64 (s, 3H), 2.60 (s, 3H), 1.28 (t, *J* = 7.6 Hz, 3H). LC-MS (ESI): *m*/*z* = 453 [M + H]^+^.

**Synthesis of K336*^a^***

*^a^*Reagents and conditions: (a) TBSCl, DMAP, DMF, r.t.; (b) LDA, TMEDA, THF, -78 °C, 20 min then CO_2_ (solid), r.t.; (c) MeI, K_2_CO_3_, DMF, r.t.; (d) TBAF, THF, r.t.; (e) MsCl, LiCl, 2,4,6-Collidine, CH_2_Cl_2_, r.t.; (f) 2, NaH, DMF, r.t.; (g) TFA, CH_2_Cl_2_, r.t.; (h) (3-bromopropoxy)-*tert*-butyldimethylsilane, NaH, DMF, r.t.; (i) 3N NaOH aq., EtOH, r.t.; (j) 3-(Aminomethyl)pyridine, EDCI・HCl, HOBt・H_2_O, DMF, r.t. K336: ^1^H-NMR (CDCl_3_) *δ*: 8.60 (d, *J* = 1.5 Hz, 1H), 8.56–8.51 (m, 1H), 7.68 (d, *J* = 7.7 Hz, 1H), 7.42 (d, *J* = 8.6 Hz, 1H), 7.30–7.23 (m, 2H), 7.16 (dd, *J* = 8.6, 1.6 Hz, 1H), 6.89 (s, 1H), 6.75 (s, 1H), 6.57 (t, *J* = 5.9 Hz, 1H), 5.53 (s, 2H), 4.65–4.57 (m, 4H), 3.60 (t, *J* = 5.7 Hz, 2H), 2.79 (q, *J* = 7.5 Hz, 2H), 2.63 (s, 3H), 2.60 (s, 3H), 2.06-1.94 (m, 2H), 1.27 (t, *J* = 7.5 Hz, 3H), 0.91 (s, 8H), 0.03 (s, 6H). LC-MS (ESI): *m*/*z* = 611 [M + H]^+^.

**Synthesis of K542, K216 and K270*^a^***

*^a^*Reagents and conditions: (a) 3N NaOH aq., EtOH, 50 °C; (b) amine, EDCI・HCl, HOBt・H_2_O, DMF, r.t.; (c) NaN_3_, DMA, 70 °C; (o) 1-pentyne, CuSO_4_・5H_2_O, sodium L-ascorbate, 1,4-dioxane, H_2_O, r.t. K542: ^1^H-NMR (DMSO-D_6_) *δ*: 11.55 (s, 1H), 8.55-8.51 (m, 1H), 8.45 (d, *J* = 1.5 Hz, 1H), 8.40 (dd, *J* = 4.8, 1.5 Hz, 1H), 7.67–7.63 (m, 1H), 7.42–7.27 (m, 3H), 7.03–6.93 (m, 3H), 5.49 (s, 2H), 3.56–3.50 (m, 2H), 2.87 (t, *J* = 7.0 Hz, 2H), 2.78 (q, *J* = 7.5 Hz, 2H), 2.51 (s, 3H), 2.50 (s, 3H), 1.19 (t, *J* = 7.5 Hz, 3H). LC-MS (ESI): *m*/*z* = 453 [M + H]^+^. K216: ^1^H-NMR (DMSO-D_6_) *δ*: 11.59 (br s, 1H), 8.50–8.45 (m, 1H), 7.66 (s, 1H), 7.42 (s, 1H), 7.35 (d, *J* = 8.6 Hz, 1H), 7.21 (s, 1H), 7.04–6.88 (m, 4H), 5.49 (s, 2H), 4.02 (t, *J* = 6.9 Hz, 2H), 3.28–3.20 (m, 2H), 2.79 (q, *J* = 7.5 Hz, 2H), 2.51 (s, 3H), 2.50 (s, 3H), 2.02–1.90 (m, 2H), 1.19 (t, *J* = 7.5 Hz, 3H). LC-MS (ESI): *m*/*z* = 456 [M + H]^+^. K270: ^1^H-NMR (DMSO-D_6_) *δ*: 11.59 (s, 1H), 8.52–8.47 (m, 1H), 7.88 (s, 1H), 7.42 (s, 1H), 7.35 (d, *J* = 8.8 Hz, 1H), 7.03–6.92 (m, 3H), 5.49 (s, 2H), 4.40–4.32 (m, 2H), 3.33–3.24 (m, 2H), 2.79 (q, *J* = 7.5 Hz, 2H), 2.58–2.44 (m, 8H), 2.10–1.99 (m, 2H), 1.62–1.54 (m, 2H), 1.19 (t, *J* = 7.5 Hz, 3H), 0.89 (t, *J* = 7.3 Hz, 3H). LC-MS (ESI): *m*/*z* = 499 [M + H]^+^.

**Synthesis of K209*^a^***

*^a^*Reagents and conditions: (a) H_2_, Pd/C, MeOH, r.t.; (b) propionic acid, PPA, 80 °C; (c) *m*CPBA, CHCl_3_, reflux; (d) POCl_3_, reflux; (e) 30% HBr/AcOH, 100 °C; (f) CO, PdCl_2_(PPh_3_)_2_, Et_3_N, MeOH, 70 °C; (g) LiAlH_4_, THF, r.t.; (h) TBSCl, imidazole, DMF, r.t.; (i) MsCl, LiCl, 2,4,6-collidine, CH_2_Cl_2_, r.t.; (j) 48, NaH, DMF, r.t.; (k) 3 N NaOH aq., EtOH, 50 °C; (l) 3-(Aminomethyl)pyridine, EDCI・HCl, HOBt・H_2_O, DMF, r.t.; (m) MsCl, Et_3_N, DMF, 0 °C (n) NaN_3_, DMA, 70 °C; (o) 1-pentyne, CuSO_4_・5H_2_O, sodium L-ascorbate, 1,4-dioxane, H_2_O, r.t. K209: ^1^H-NMR (CDCl_3_) *δ*: 9.19 (s, 1H), 8.66–8.52 (m, 2H), 7.70 (d, *J* = 7.9 Hz, 1H), 7.40–7.23 (m, 4H), 7.13 (dd, *J* = 8.6, 1.5 Hz, 1H), 6.97 (s, 1H), 6.78 (d, *J* = 1.5 Hz, 1H), 6.70–6.63 (m, 1H), 5.64 (s, 2H), 5.51 (s, 2H), 4.68 (d, *J* = 6.0 Hz, 2H), 2.87 (q, *J* = 7.5 Hz, 2H), 2.66–2.57 (m, 5H), 2.25–2.16 (m, 2H), 1.32 (t, *J* = 7.5 Hz, 3H), 0.88 (t, *J* = 7.2 Hz, 3H). LC-MS (ESI): *m*/*z* = 548 [M + H]^+^.

**Synthesis of Ind-Tag and Bnz-Tag*^a^***

**Procedure 1:**

To [NH_2_-CH_2_CH_2_-(O-CH_2_CH_2_)_6_CO-Asp(tBu)-Tyr(tBu)-Lys(Boc)-Asp(tBu)-Asp(tBu)-Asp(tBu)-Asp(tBu)-Lys(Boc)-resin] (0.0584 g) prepared by the reported procedure (Patent: WO2005094187) was added DMF (0.300 mL), HBTU (0.106 g, 0.281 mmol), HOBT･H_2_O (0.040 g, 0.263 mmol), *N*,*N*-diisopropylethylamine (0.098 mL, 0.561 mmol) and 4-penthinic acid (0.028 g, 0.281 mmol). The mixture was incubated overnight at room temperature. The resin was washed by DMF (1 mL, 5 times), methanol (1 mL, 5 times) and dibuthylether (1 mL, 5 times). To the residue was added 90% trifluoroacetic acid solution (0.6 mL), and the mixture was incubated for 2 h at room temperature. After filtration, diethy ether (14 mL) was added to the filtrate, and the solution was centrifuged (3000 rpm) for 5 mins. After decantation, the residue was dried under reduced pressure to give the target material (0.041 g, 0.029 mmol). LC-MS (ESI): *m*/*z* = 715 [M + 2H]^2+^, 1429 [M + H]^+^.

**Procedure 2:**

To a solution of compound 51 (3.0 mg, 6.26 µmol) in 1,4-dioxane (0.8 mL) was added H_2_O (0.178 mL), CuSO_4_・5H_2_O, (7.81 µg, 0.0313 µmol), sodium L-ascorbate (12.0 µg, 0.0606 µmol) and compound 50 (15.2 mg, 10.6 µmol). The mixture was stirred overnight at room temperature. The reaction mixture was diluted by H_2_O (0.250 mL) and purified by preparative HPLC to give the target material (5.1 mg, 43%). HR-LC-MS (ESI): m/z for C_87_H_119_N_20_O_29_ [M + 2H]^2+^ : calcd. 954.4259, found 954.4236.

**Procedure 3:**

The target material (5.0 mg, 26%) was prepared from compound 15 (5.1 mg, 9.99 µmol) according to the same method described in procedure 2. HR-LC-MS (ESI): m/z for C_88_H_120_N_19_O_31_ [M + 2H]^2+^ : calcd. 969.92315, found 969.9202.

**Supplemental Figures**


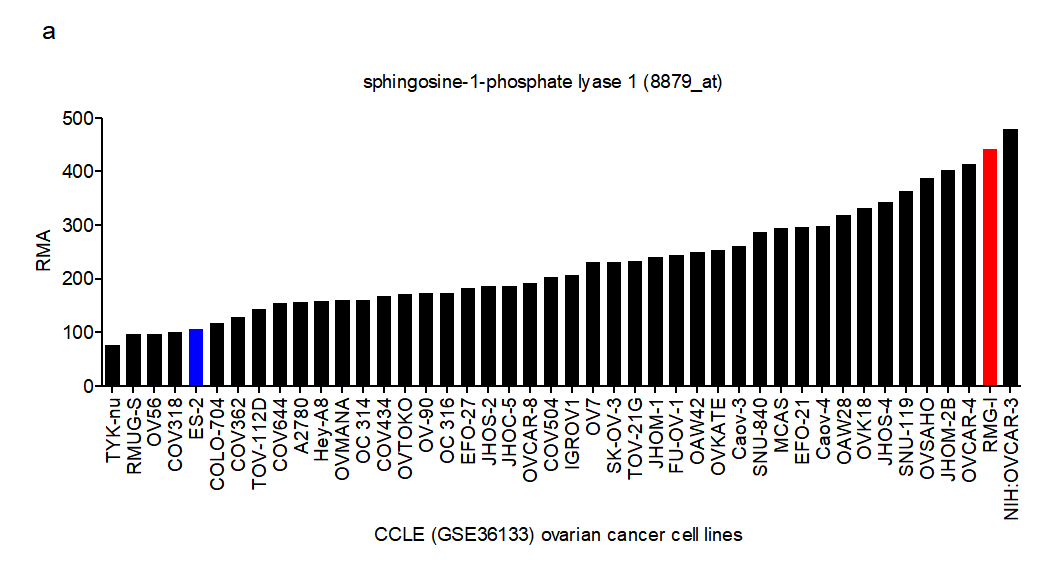


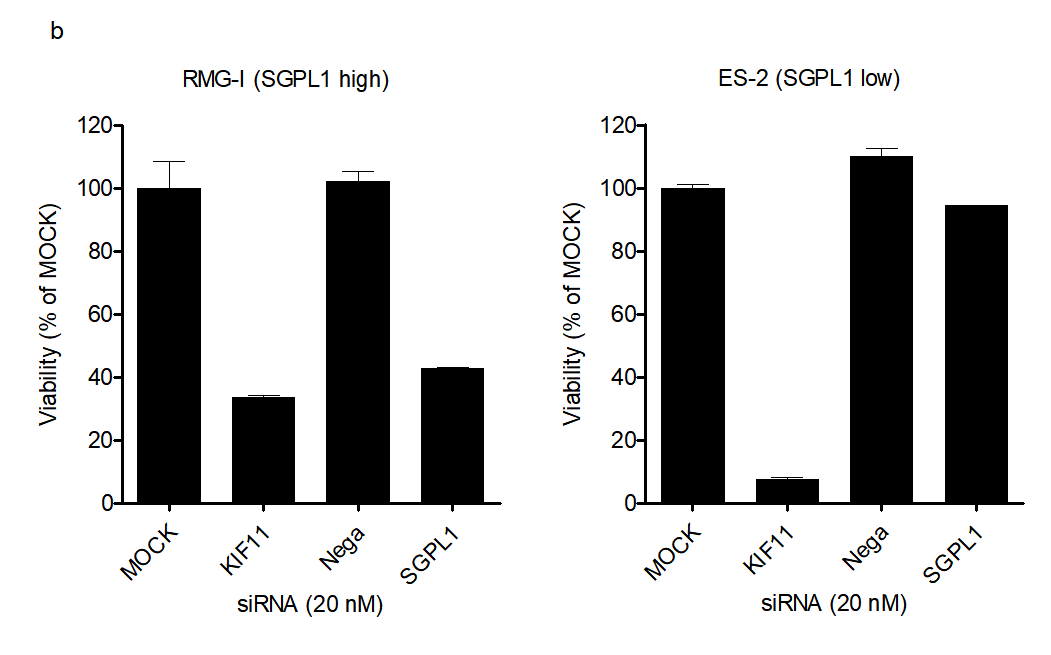


**Supplemental Fig. S1. The SGPL-1 expression in ovarian cancer cell lines and the cytotoxic activity of SGPL1-targeting siRNA on ovarian cancer cells.** a) The SGPL1 mRNA expression in various ovarian cancer cell lines analyzed in a public database. The expression data were extracted from NCBI Gene Expression Omnibus (GEO): GSE36133 (<https://www.ncbi.nlm.nih.gov/geo/query/acc.cgi?acc=GSE36133>) ^ref.1^ , and the bar graph was generated using the Prism software program. The expression of SGPL1 in ES-2 and RMG-I cells is represented with blue and red bars, respectively. b) The cytotoxic activity of SGPL1 siRNA on RMG-I and ES-2 cells. RMG-I and ES-2 cells were treated with 20 nM siRNA for KIF11 (positive control of cytotoxity), SGPL1 or Nega (non-targeting control) for 72 h. The cell viability was measured using a WST assay. Data indicate the mean±s.e.m., *n* = 3.


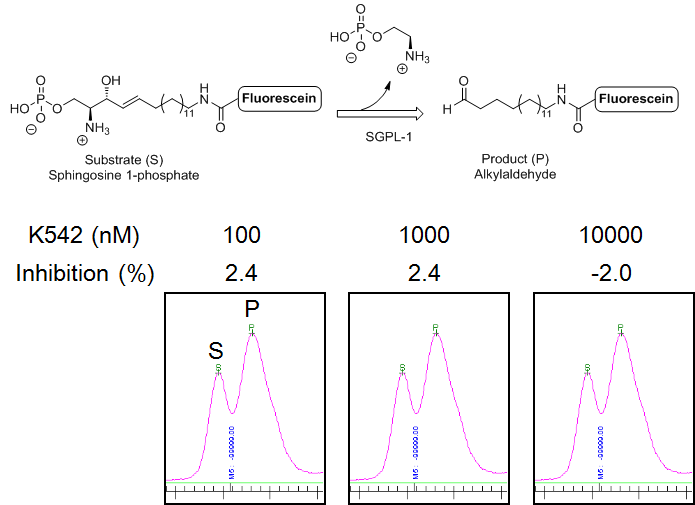


**Supplemental Fig. S2. The SGPL1 enzyme assay**

A schematic representation of the SGPL1 enzyme assay is shown in the upper figure. Substrate (S) and Product (P) were separated by a mobility shift assay using an EZ reader. K542 did not shown any inhibition of the SGPL-1 enzyme activity.


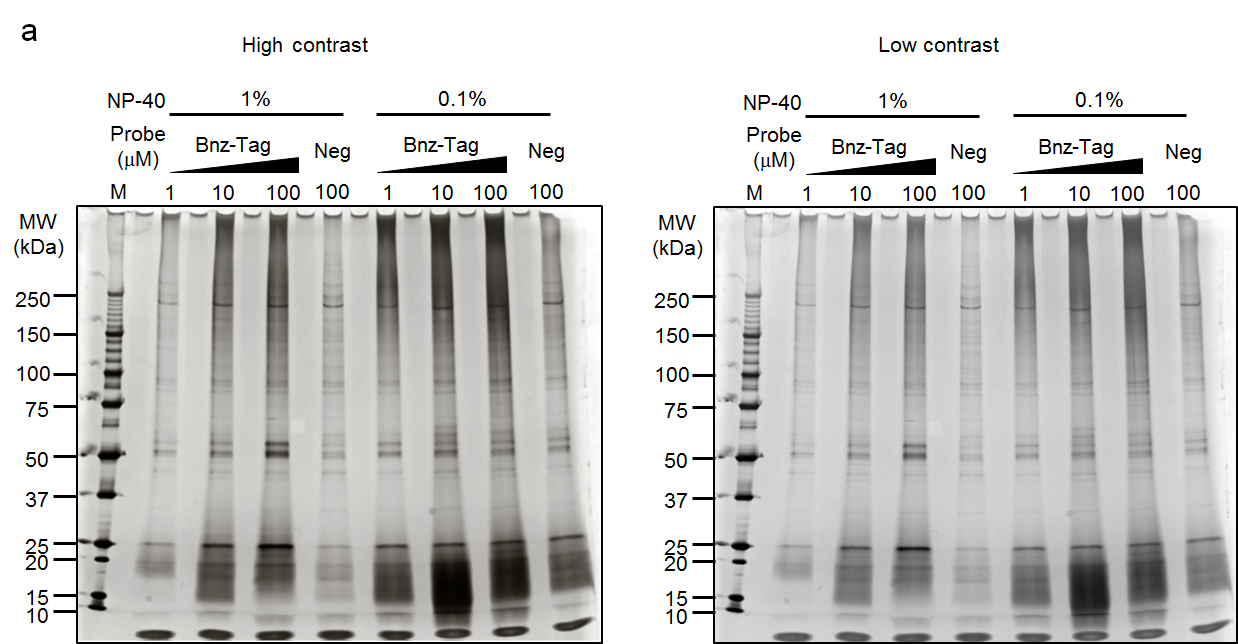

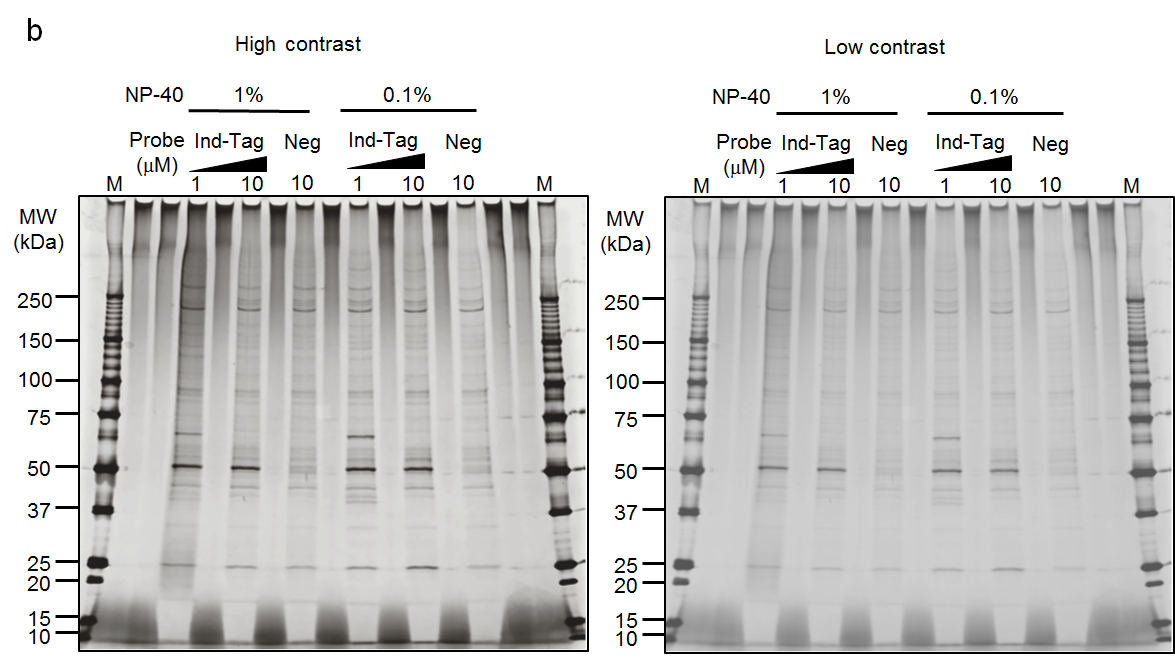
**Supplemental Fig. S3. The chemical pull-down experiment using Ind-Tag and the Bnz-Tag affinity probe.**

a) Pulldown samples were prepared using Bnz-Tag at indicated concentration and separated on SDS-PAGE. Original full gel images detected with silver staining using the trans-illumination mode of an LAS4000 are shown in multiple contrast images. Neg means the experiment was performed using a negative control probe. No significant or specific Bnz-Tag-binders were observed. b) Original full gel images for Figure 2A detected with silver staining using trans-illumination mode of LAS4000 are shown in multiple contrast images.


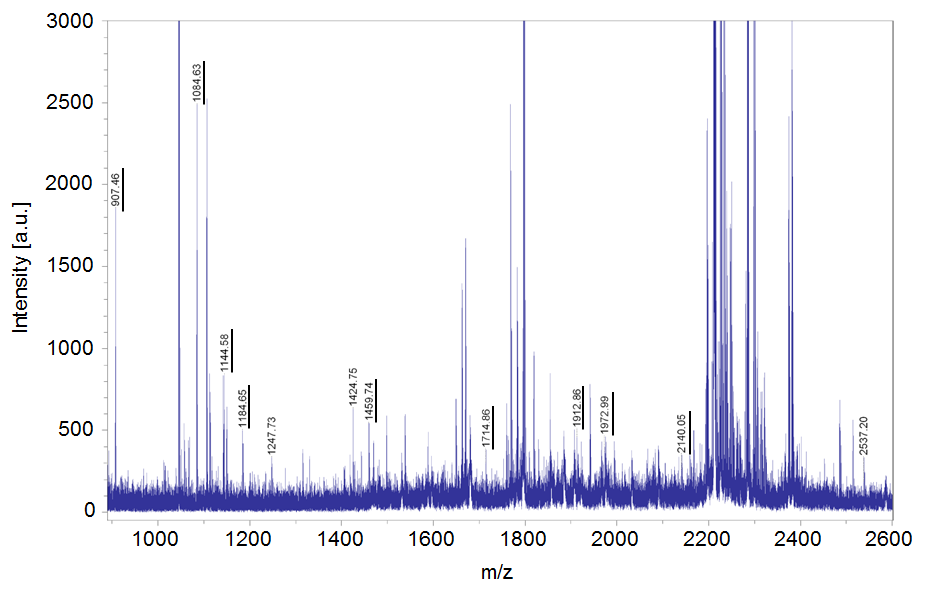


**Supplemental Fig. S4. The MALDI-TOF-MS spectra of the 50-kDa protein binding to Ind-Tag in Figure 2A.**

The underlined ions were assigned in NAMPT (P43490).


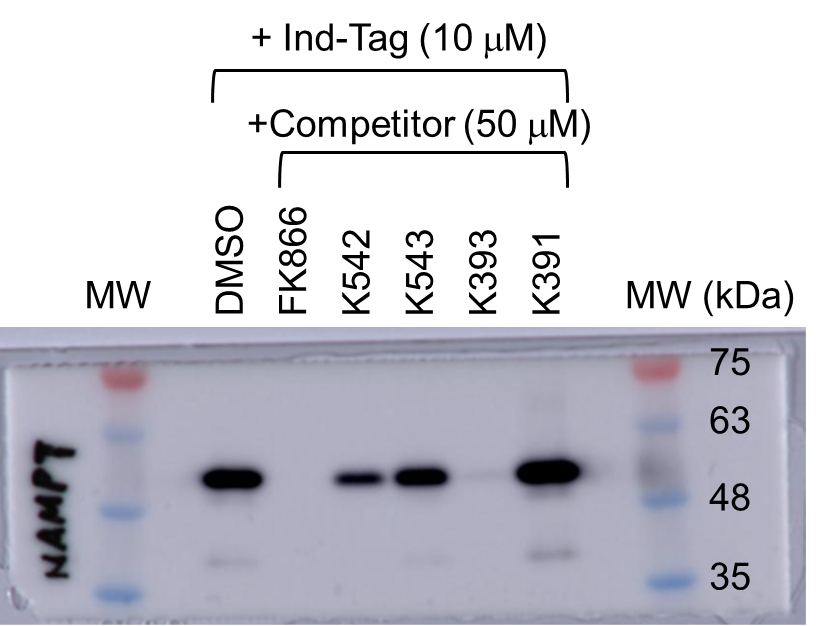


**Supplemental Fig. S5. The competitive pull-down experiment using the Ind-Tag and various competitors.**

A detected full-blot image for Figure 2C. The chemiluminescence image was merged with the colorimetric epi-illumination image.


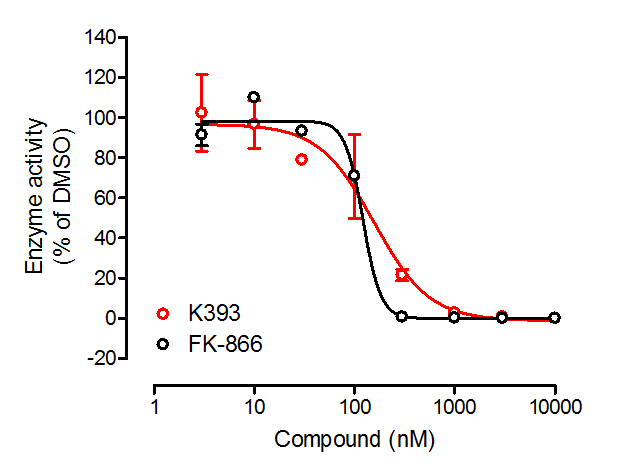


**Supplemental Fig. S6. An enzyme inhibition assay of NAMPT inhibitors.**

NAMPT enzyme was treated with various concentrations of the potent inhibitor, K393 (red) and the known NAMPT inhibitor, FK-866 (black). NAD^+^ production was measured with a colorimetric assay. Data indicate the mean ± s.e.m., *n* = 2.


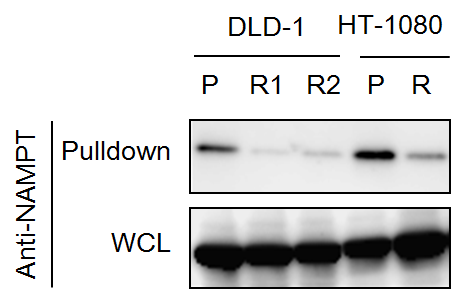


**Supplemental Fig. S7. A NAMPT pull-down assay using K542-resistant cells.**

Resistant DLD-1(R1 and R2) and resistant HT-1080 (R) expressed NAMPT protein at the same level as the respective parent cell (P). In the pull-down fraction, the probe-binding NAMPT was markedly reduced in resistant cells. Each of the pulldown and WCL (Whole Cell Lysate) blots were individually detected with chemiluminescence and cropped from different blots.


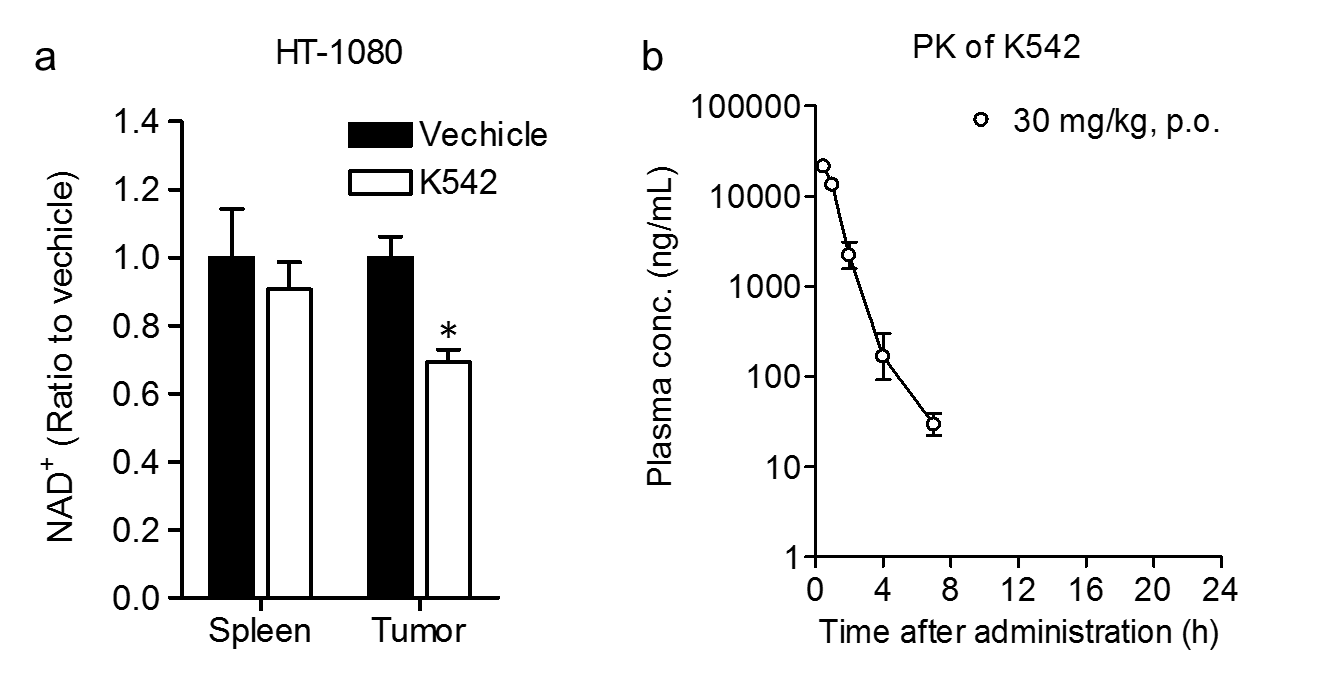


**Supplemental Fig. S8. The PK profile and intratumoral NAMPT inhibition activity of K542.**

a) HT-1080 xenograft models were orally treated with vehicle or K542 (30 mg/kg, twice daily) for 4.5 days, and tumors and tissues were harvested 8 h after the final administration for the measurement of NAD+ by LC-MS/MS. K542 was not detected at 24 hours after treatment. Data indicate the mean ± s.e.m., *n* = 3. *p < 0.05, Student’s *t*-test. b) BALB/cAnNCrlCrlj mice were orally treated with K542 once (30 mg/kg). After administration, blood samples were collected from the tail vein and the plasma concentration of K542 was measured by LC/MS-MS. Data indicate the mean ± s.e.m., *n* = 2.

**Reference**

1. Barretina, J*. et a*l. The Cancer Cell Line Encyclopedia enables predictive modelling of anticancer drug sensitivity*. Natu*r**e 48**3, 603–307 (2012).
